# Overexpression of native carbonic anhydrases increases carbon conversion efficiency in the methanotrophic biocatalyst *Methylococcus capsulatus* Bath

**DOI:** 10.1128/msphere.00496-24

**Published:** 2024-08-27

**Authors:** Spencer A. Lee, Jessica M. Henard, Robyn A. C. Alba, Chance A. Benedict, Tyler A. Mayes, Calvin A. Henard

**Affiliations:** 1 BioDiscovery Institute and Department of Biological Sciences, University of North Texas, Denton, Texas, USA; E. O. Lawrence Berkeley National Laboratory, Berkeley, California, USA

**Keywords:** methanotroph, carbonic anhydrase, C1 metabolism, methane, carbon dioxide, greenhouse gas

## Abstract

Methanotrophic bacteria play a vital role in the biogeochemical carbon cycle due to their unique ability to use CH_4_ as a carbon and energy source. Evidence suggests that some methanotrophs, including *Methylococcus capsulatus*, can also use CO_2_ as a carbon source, making these bacteria promising candidates for developing biotechnologies targeting greenhouse gas capture and mitigation. However, a deeper understanding of the dual CH_4_ and CO_2_ metabolism is needed to guide methanotroph strain improvements and realize their industrial utility. In this study, we show that *M. capsulatus* expresses five carbonic anhydrase (CA) isoforms, one α-CA, one γ-CA, and three β-CAs, that play a role in its inorganic carbon metabolism and CO_2_-dependent growth. The CA isoforms are differentially expressed, and transcription of all isoform genes is induced in response to CO_2_ limitation. CA null mutant strains exhibited markedly impaired growth compared to an isogenic wild-type control, suggesting that the CA isoforms have independent, non-redundant roles in *M. capsulatus* metabolism and physiology. Overexpression of some, but not all, CA isoforms improved bacterial growth kinetics and decreased CO_2_ evolution from CH_4_-consuming cultures. Notably, we developed an engineered methanotrophic biocatalyst overexpressing the native α-CA and β-CA with a 2.5-fold improvement in the conversion of CH_4_ to biomass. Given that product yield is a significant cost driver of methanotroph-based bioprocesses, the engineered strain developed here could improve the economics of CH_4_ biocatalysis, including the production of single-cell protein from natural gas or anaerobic digestion-derived biogas.

**IMPORTANCE:**

Methanotrophs transform CH_4_ into CO_2_ and multi-carbon compounds, so they play a critical role in the global carbon cycle and are of interest for biotechnology applications. Some methanotrophs, including *Methylococcus capsulatus*, can also use CO_2_ as a carbon source, but this dual one-carbon metabolism is incompletely understood. In this study, we show that *M. capsulatus* carbonic anhydrases are critical for this bacterium to optimally utilize CO_2_. We developed an engineered strain with improved CO_2_ utilization capacity that increased the overall carbon conversion to cell biomass. The improvements to methanotroph-based product yields observed here are expected to reduce costs associated with CH_4_ conversion bioprocesses.

## INTRODUCTION

Methanotrophic bacteria (methanotrophs) use methane (CH_4_) as both a carbon and energy source. These unique microbes are ubiquitous in terrestrial and aquatic ecosystems where they play a vital role in the biogeochemical cycling of CH_4_, a potent greenhouse gas (GHG). Due to their unique metabolism, methanotrophs are used industrially for the conversion of CH_4_-rich gas to valuable products, including single-cell protein and polyhydroxyalkanoates (1, 2). Furthermore, methanotrophs may be leveraged for direct air capture of CH_4_ for GHG mitigation and combating climate change (3, 4).

In these bacteria, CH_4_ assimilation is mediated by cytoplasmic- or membrane-associated CH_4_ monooxygenases (MMO), which use molecular oxygen to oxidize CH_4_ to methanol (CH_3_OH) (5, 6). CH_3_OH is further oxidized to formaldehyde (CH_2_O) in the periplasm by calcium- or lanthanide-dependent CH_3_OH dehydrogenases. The CH_2_O can enter central metabolism in the cytosol by (i) directly condensing with a C_5_ sugar in the ribulose monophosphate (RuMP) cycle, (ii) conversion to methylene tetrahydrofolate (H_4_F) *via* methylene tetrahydromethanopterin- and H_4_F-C_1_ transfer pathways followed by condensation with glycine in the serine cycle, or (iii) complete oxidation to carbon dioxide (CO_2_), which can be assimilated *via* autotrophic pathways (7). Methanotrophs are broadly categorized based on their preferred route of CH_2_O metabolism, wherein Gammaproteobacterial methanotrophs use the RuMP cycle, Alphaproteobacterial methanotrophs use the serine cycle, and Verrucomicrobia/Candidate phylum NC10 methanotrophs use the ribulose-1,5-bisphophate carboxylase/oxygenase (RubisCO) and the Calvin cycle to reassimilate CH_4_-derived CO_2_ (8). Notably, all aerobic methanotrophs completely oxidize a percentage (Gammaproteobacteria and Alphaprotebacteria) or all (Verrucomicrobia/Candidate phyla NC10) of assimilated CH_4_ to CO_2_ (9–11).

*Methylococcus capsulatus* str. Bath is a model methanotroph that has served to elucidate several fundamental aspects of methanotrophy (12), including structural elucidation of the particulate MMO (6, 13, 14), and characterization of the generalizable “copper switch” physiological response to copper starvation in methanotrophs that encode both the soluble and particulate MMO forms (15, 16), among others. *M. capsulatus* belongs to a phylogenetical distinct lineage of methanotrophs within the *Methylococcaceae* family of Gammaproteobacterial methanotrophs (Type Ib) that includes the *Methylocaldum*, *Methylococcus*, *Methylogaea*, *Methylomagnum*, and *Methyloterricola* genera (17). These methanotrophs encode RubisCO, but, in contrast to Verrucomicrobia, *M. capsulatus* derives its biomass from CH_4_-derived formaldehyde *via* the RuMP cycle and CO_2_ *via* RubisCO (18). Previous studies show that *M. capsulatus* RubisCO is expressed and active (19–21) and that both the enzyme and CO_2_ are essential for bacterial cultivation (18).

Most RubisCO enzymes exhibit poor efficiency with low catalytic rate constants and substrate specificity (22). Thus, many autotrophic bacteria have developed inorganic carbon concentrating mechanisms (CCMs) to improve CO_2_ assimilation (23). One of the most characterized CCMs are carboxysomes in photoautotrophic cyanobacteria, which are proteinaceous “organelles” that house RubisCO, a bicarbonate transporter, and carbonic anhydrase (24). Genes encoding the carboxysome structural shell proteins are not found in the *M. capsulatus* genome and carboxysomes are not visualized in electron micrographs, providing strong evidence that *M. capsulatus* does not encode these structures to enhance CO_2_ assimilation. CCMs in non-phototrophic autotrophic bacteria without carboxysomes are not well understood. *M. capsulatus* is cultivated aerobically and relatively low concentrations of CO_2_ in a CH_4_-rich gas mixture permit growth (18), so this methanotroph may possess alternative CCMs to enhance RubisCO CO_2_ substrate specificity.

CAs are metalloenzymes that mediate the reversible hydration of CO_2_ to H^+^ and bicarbonate (HCO_3_^-^) and are represented in all domains of life (25). These enzymes typically have a substrate preference for either CO_2_ or HCO_3_^−^, which can promote CO_2_ conversion or production depending on the environmental condition (25). Bacteria typically encode one or more of the α-, β-, and/or γ-classes of CAs, which contribute to their survival and adaptation in diverse environments through their roles in pH homeostasis, metabolic pathway regulation, ion transport, and CO_2_ fixation and conversion (25–27). In many autotrophic bacteria, CAs mediate a CCM by sequestering CO_2_ and converting it to HCO_3_^−^, which does not readily diffuse from the cell (28). In turn, intracellular CAs convert HCO_3_^−^ to CO_2_ colocalized with RubisCO, increasing substrate availability and conversion efficiency (29, 30). CAs have been successfully utilized to enhance carbon capture and growth of engineered microbes (31–36), underscoring their biotechnology potential to sequester atmospheric CO_2_ and mitigate GHGs.

In this study, we used a genetics approach to evaluate the role(s) of five annotated CAs in *M. capsulatus* metabolism. We show that the five distinct CA isoforms are transcribed and exhibit differential induction in response to CO_2_ availability. Experiments evaluating the pharmacological inhibition of *M. capsulatus* CAs and the growth of strains deficient in individual CAs provide evidence that these enzymes play critically important, non-redundant roles in *M. capsulatus* metabolism and physiology. Overexpression of CAs improved *M. capsulatus* growth kinetics and CH_4_ conversion efficiencies. Our results advance the understanding of inorganic carbon metabolism in methanotrophic bacteria and highlight a genetic engineering strategy that leverages CAs to mitigate both CH_4_ and CO_2_ GHGs.

## MATERIALS AND METHODS

### Bacterial cultivation

Bacterial strains used in this study are shown in Table 1. *M. capsulatus* Bath cultures were routinely maintained with nitrate mineral salts (NMS) solid medium in stainless steel gas chambers supplied with 20% CH_4_ in the gas phase as previously described (37). Strains were grown in 150 mL vials containing 10  mL of liquid NMS medium or 250 mL vials containing 20 mL of liquid NMS medium. After inoculation with plate-derived biomass or diluted seed culture, vials were crimped with gray butyl stoppers to create gas-tight seals. CH_4_ was added to the headspace to reach a final CH_4_ concentration of 20% in the air (vol/vol), and cultures were incubated at 37°C at 200 rpm orbital shaking. Continuous gas cultivation was performed using a custom mid-throughput gas fermentation reactor fitted with 150 mL, 38 mm Kimax cultivation tubes supplied with mixed gas *via* rotameters and stainless steel sparge stones. 100 mL cultures were inoculated with plate-derived biomass to OD_600_ = 0.1 and supplied with 20% CH_4_/2% CO_2_ in air (vol/vol) at a flow rate of 1 vol gas/volume culture/min (vvm) premixed with gas-specific mass flow controllers. Culture samples (~100 µL) were periodically extracted from vials with a syringe to measure bacterial growth *via* dilution plating or spectrophotometrically at OD_600_ using a Nanodrop spectrophotometer. High-resolution bacterial growth was measured every 20 seconds by Cell Growth Quantifier (CGQ) optical sensors (Scientific Bioprocessing) to determine growth rates. The backscatter arbitrary units measured by the CGQ sensors were transformed to OD_600_ using linear regression of measured initial and final OD_600_ measurements. The CGQ sensor limit of detection is equivalent to OD_600_ 0.5.

**TABLE 1:** Strains and plasmids

Strains		
Name	Genotype	Source
*Methylococcus capsulatus* str. Bath	Wild type	Lab Stock
Δ*CA1::Kn^R^*	MCA0910 gene replaced with an FRT-flanked Gm^R^ cassette	This study
Δ*CA2::Gm^R^*	MCA1080 gene replaced with an FRT-flanked Kn^R^ cassette	This study
Δ*CA3::Gm^R^*	MCA1422 gene replaced with an FRT-flanked Gm^R^ cassette	This study
Δ*CA4::Kn^R^*	MCA1665 gene replaced with an FRT-flanked Kn^R^ cassette	This study
Δ*CA5::Gm^R^*	MCA2797 gene replaced with an FRT-flanked Gm^R^ cassette	This study
pCA1 Bath	*M. capsulatus* with inducible overexpression of MCA0910	This study
pCA2 Bath	*M. capsulatus* with inducible overexpression of MCA1080	This study
pCA3 Bath	*M. capsulatus* with inducible overexpression of MCA1422	This study
pCA4 Bath	*M. capsulatus* with inducible overexpression of MCA1665	This study
pCA5 Bath	*M. capsulatus* with inducible overexpression of MCA2797	This study
pCA2/3 Bath	*M. capsulatus* with inducible overexpression of MCA1080 and MCA1422	This study
pCA1 Δ*CA1::Gm^R^*	*M. capsulatus***Δ***CA1::Gm^R^* with inducible overexpression of MCA0910	This study
pCA3 Δ*CA3::Gm^R^*	*M. capsulatus***Δ***CA3::Gm^R^* with inducible overexpression of MCA1422	This study
pCA5 Δ*CA5::Gm^R^*	*M. capsulatus***Δ***CA5::Gm^R^* with inducible overexpression of MCA2797	This study
*Escherichia coli* str. Zymo 10B	F- *mcrA* ∆(*mrr-hsdRMS-mcrBC*) Φ80*lacZ*∆M15 ∆*lacX*74 *recA1 endA1 araD139* ∆(*ara leu*) 7697 *galU galK rpsL nupG*	Zymo Research
*E. coli* S17-1	Tp^r^ Sm^r^ *recA thi pro hsd*(r^-^m^+^)RP4-2-Tc::Mu::Km Tn7	ATCC 47055

Plasmids		
Name	Description	Source
pCAH01	P*_tetA_ bla tetR* CoE1*ori* F1 *oriV oriT trfA ahp*	(38)
pCA1	pCAH01 plasmid with *M. capsulatus* CA1 (MCA0910)	This study
pCA2	pCAH01 plasmid with *M. capsulatus* CA2 (MCA1080)	This study
pCA3	pCAH01 plasmid with *M. capsulatus* CA3 (MCA1422)	This study
pCA4	pCAH01 plasmid with *M. capsulatus* CA4 (MCA1665)	This study
pCA5	pCAH01 plasmid with *M. capsulatus* CA5 (MCA2797)	This study
pCA2/3	pCAH01 plasmid with *M. capsulatus* CA2 (MCA1080) and CA3 (MCA1422) in an operon.	This study
pK18mobsacB	Marker-exchange mutagenesis suicide plasmid with *sacB* counterselectable marker	(39)
pK18mobpheS	Marker-exchange mutagenesis suicide plasmid with *pheS* counterselectable marker	This study
pPS856	Source of FRT-flanked *aacC* Gm^R^ cassette	(40)
pKD13	Source of FRT-flanked *aph* Kn^R^ cassette	(41)

### Carbonic anhydrase knockout and overexpression strain construction

Primers used for cloning and genetic manipulations are shown in Table 2. All PCRs were performed with Q5 High-Fidelity 2X Master Mix following the manufacturer’s recommendations (New England Biolabs), and PCR annealing temperatures were determined using the NEB Tm Calculator tool. Plasmids were assembled using NEBuilder HiFi DNA Assembly Master Mix following the manufacturer’s vector:insert molar ratio recommendations, but total reaction volumes were minimized and typically between 4 and 10 μL, depending on the amount of assembled fragments (New England Biolabs). *M. capsulatus* Bath carbonic anhydrase (MCA0910, MCA1080, MCA1422, MCA1665, and MCA2797) knock-out strains were created by amplifying 1,000 bp DNA regions upstream and downstream flanking the genes of interest from genomic DNA and an Flp recombinase recognition target (FRT)-flanked gentamicin or kanamycin resistance cassette from pPS856 or pKD13, respectively. The 1 kb upstream-antibiotic resistance-1kb downstream was assembled with pK18mobpheS linearized *via* PCR using primers oCAH576/577 and transformed into chemically competent *E. coli* DH10b. Sequence-confirmed plasmids were then transferred into S17-1 *E. coli via* chemical transformation to mediate biparental conjugation with *M. capsulatus* as previously described (42). *M. capsulatus* transformants were selected on NMS containing 50 µg/mL kanamycin or 20 µg/mL gentamicin and 10 mM 4-chloro-phenylalanine to select for double-crossover events. Antibiotic- and 4-chloro-phenyalanine-resistant transformant colonies were screened by PCR to confirm the replacement of a CA locus with the antibiotic resistance cassette. Notably, antibiotic resistant, small, atypical CA KO transformant colonies typically appeared 4 weeks [compared to 1 week for wild type (WT)] after plating on a selective medium. The pK18mobpheS plasmid was generated following methods found in Ishikawa et al. (43). Here, a *M. capsulatus*-mutated *pheS* gene (minimally codon-optimized to enable synthesis) encoding the α-subunit of phenylalanyl-tRNA synthetase containing two missense mutations (A306G and T252A) was synthesized as a gblock by Integrated DNA Technologies and assembled with pK18mobsacB linearized with primers oCAH572/oCAH573 to replace *sacB* with the mutated *pheS* gene.

**TABLE 2:** Synthetic DNA and primers^*a*^

Fragment/Primer name	Sequence	Purpose
*M. capsulatus* codon-optimized *pheS* T252A /A306G	gtacataaaaaaggagacatgaacgATGAACTCGTCGCCCGAATCCACCCTGGAGCAGGCGAGCAGGCTGCTGGCCGCCGCGGGAAGCGTGGCAGAATTAGACCAGGTACGCGTCCGCTATCTGGGGAAGAAGGGCGAGTTCACCGAGCAGATGAAGACGCTGGGCACGCTCTCGCCGGAAGAACGCAAGGAATTTGGTCAGCGCGTCAATCAGGCACGTGACGAGTTCCAGCGGCTCCTGGAGCGCCGCAAGGCGGCGCTGGAAGCCGAAGCCCTGGCACGGCGGCTGAGTAGCGAGACGATCGATGTCACGCTCCCTGGCCGTGGGCAGCGACTGGGAGGACTGCACCCGGTCACGTTAACCCTCCGACGCATCACCAGGCTGTTCCGGTCAGTTGGCTTCAGCGTCGTGGAAGGCCCGGAGATTGAAGACGACTTTCATAACTTCGAAGCCTTGAATATTCCCGCCCACCATCCGGCGCGCGCGATGCATGACACATTCTACTTCTCCGAACATCTCCTCCTGCGTACCCACACCAGCCCGGTCCAGATCCGCGTTATGGAGTCCGGGCAGCCCCCGCTCCGGGTGATAGCCCCCGGACGGGTCTATCGGTGCGACAGCGACCTGACCCACACGCCGATGTTTCACCAGGTCGAGGGCTTCTGGGTTGATGAGCAGGTGTCGTTCGCCGATCTGAAGGGCACGTTGTATGAGTTCCTTACCGGCTTCTTCGAGAAGGACTGTGCCGTACGGTTCCGGCCGAGCTACTTCCCGTTTGCGGAACCCTCGGCCGAGGTCGACATCGAGTGCGTCATCTGCGATGGTCGGGGCTGCCGGGTATGCAAGCATTCAGGCTGGCTGGAGGTGATGGGCTGTGGCATGATCCATCCCCGCGTCTTCGAGGCCGTGGGCATCGACCCCGAACGGTATTCGGGCTTCGGCTTCGGCCTGGGCGTCGAGCGCCTGACGATGCTGCGCTACGGCATCAACGACCTTCGCCTCTTCTTCGAGAATGATCTGCGCTTCCTGCGCCAGTTCAAGCCCTTCTGAaaaacgcaaagaaaatgccgatggg	gblock used to generate pK18mobpheS marker exchange mutagenesis plasmid
*M. capsulatus carbonic anhydrase (CA) knockouts*		
CAH723 MCA1442KO 1kbUP F	acagctatgacatgattacgaattcTCGATGGCGGGATCGTCTTG	Amplify 1 Kb homology from *M. capsulatus* upstream of MCA1442
CAH063 MCA1442KO 1kbUP R	gatccccaattcgGAGTTCGCTCTTAGGTTTTGAAAGG	
CAH064 FRT-Gm F	aagagcgaactcCGAATTGGGGATCTTGAAG	Amplify 1.2 Kb Gm^R^ cassette from pPS856
CAH065 FRT-Gm R	caggtcagccgcCGAATTAGCTTCAAAAGC	
CAH066 MCA1442KO 1kbDN F	tgaagctaattcgGCGGCTGACCTGGTCTTC	Amplify 1 Kb homology from *M. capsulatus* downstream of MCA1442
CAH724 MCA1422KO 1kbDN R	taaaacgacggccagtgccaagcttTCTCCGGTGGATGCCGCC	
CAH129 MCA1442KO flank F	CCGCCTTTCCCTTTCAAAACC	Confirm MCA1442 knockout
CAH130 MCA1442KO flank R	TGAAGACCAGGTCAGCCG	
CAH725 MCA1665KO 1kbUP F	acagctatgacatgattacgaattcATATGTCACAATCCGCTTC	Amplify 1 Kb homology from *M. capsulatus* upstream of MCA1665
CAH069 MCA1665KO 1kbUP R	agctccagcctacacGATTTCTGTCCTTGATAAGG	
CAH070 FRT-Kn F	aggacagaaatcGTGTAGGCTGGAGCTGCTTC	Amplify 1 Kb Kn^R^ cassette from pKD13
CAH071 FRT-Kn R	acaggcaagaaCTGTCAAACATGAGAATTAATTCCGG	
CAH072 1665KO 1kbDN F	tctcatgtttgacagTTCTTGCCTGTGACCGATTG	Amplify 1 Kb homology from *M. capsulatus* downstream of MCA1665
CAH726 1665KO 1kbDN R	taaaacgacggccagtgccaagcttATGTGGCGGCTAGTACAG	
CAH131 MCA1665KO flank F	GCATCCACCTTATCAAGGACAGAA	Confirm MCA1665 knockout
CAH132 MCA1665KO flank R	TTCTCGGGCCGCTTCATC	
CAH727 MCA2797KO 1kbUP F	acagctatgacatgattacgaattcATCCTCGATTTCGAAGATGG	Amplify 1 Kb homology from *M. capsulatus* upstream of MCA2797
CAH075 MCA2797KO 1kbUP R	gatccccaattcgCTTTCCTCTCTGCCGGTC	
CAH076 FRT-Gm F	cagagaggaaagCGAATTGGGGATCTTGAAG	Amplify 1.2 Kb Gm^R^ cassette from pPS856
CAH077 FRT-Gm R	gcgcatgttgggCGAATTAGCTTCAAAAGC	
CAH078 MCA2797KO 1kbDN F	tgaagctaattcgCCCAACATGCGCAAGACTC	Amplify 1 Kb homology from *M. capsulatus* downstream of MCA2792
CAH728 MCA2797KO 1kbDN R	taaaacgacggccagtgccaagcttCGTGACGGTGACGTTATTG	
CAH133 MCA2797KO flank F	CGACCGGCAGAGAGGAAAG	Confirm MCA2797 knockout
CAH134 MCA2797KO flank R	GAGTCTTGCGCATGTTGGG	
CAH729 MCA0910KO 1kbUP F	acagctatgacatgattacgaattcGCCCTGCATGTATGAACC	Amplify 1 Kb homology from *M. capsulatus* upstream of MCA0910
CAH081 MCA0910KO 1kbUP R	agctccagcctacacCGTGTATATCTCCTCTGGG	
CAH082 FRT-Kn F	gagatatacacgGTGTAGGCTGGAGCTGCTTC	Amplify 1 Kb Kn^R^ cassette from pKD13
CAH083 FRT-Kn R	ggtcgccacggcCTGTCAAACATGAGAATTAATTCCGG	
CAH084 MCA0910KO 1kbDN F	tctcatgtttgacagGCCGTGGCGACCTCGAAAG	Amplify 1 Kb homology from *M. capsulatus* downstream of MCA0910
CAH730 MCA0910KO 1kbDN R	taaaacgacggccagtgccaagcttCCCTGGTCGAAGCAGGCG	
CAH135 MCA0910 flank F	ACACAACCCAGAGGAGATATACACG	Confirm MCA0910 knockout
CAH136 MCA0910 flank R	TGATTGGCCTCTGCGAAGAAA	
CAH731 MCA1080KO 1kbUP F	acagctatgacatgattacgaattcCGTCCCGCAGTTCCGCCG	Amplify 1 Kb homology from *M. capsulatus* upstream of MCA1080
CAH087 MCA1080KO 1kbUP R	gatccccaattcgCCCAATTCCTCCCTTCAACGGC	
CAH088 FRT-Gm F	ggaggaattgggCGAATTGGGGATCTTGAAG	Amplify 1.2 Kb Gm^R^ cassette from pPS856
CAH089 FRT-Gm R	gacgattcgtgaCGAATTAGCTTCAAAAGC	
CAH090 MCA1080KO 1kbDN F	tgaagctaattcgTCACGAATCGTCCCCTCC	Amplify 1 Kb homology from *M. capsulatus* downstream of MCA1080
CAH732 MCA1080KO 1kbDN R	taaaacgacggccagtgccaagcttGGCGATCTTATTTGCTCGAAG	
CAH137 MCA1080 flank F	CCGTTGAAGGGAGGAATTGGG	Confirm MCA1080 knockout
CAH138 MCA1080 flank R	TTTGGCCTGCGCGTTAAAC	
CAH572 pK18mobsacB F	AAACGCAAAAGAAAATGC	Linearize pK18mobsacB for pK18mobpheS construction
CAH573 pK18mobsacB R	CGTTCATGTCTCCTTTTTTATG	
CAH576 pK18mobpheS F	AAGCTTGGCACTGGCCGT	Linearize pK18mobpheS for knockout construction
CAH577 pK18mobpheS R	GAATTCGTAATCATGTCATAGCTGTTTCCTG	
CAH618 pK18mobpheS seq F	TGTTGTGTGGAATTGTGAGC	Sequencing primers for pK18mobpheS constructs
CAH619 pK18mobpheS seq R	GATGTGCTGCAAGGCGATTA	
*M. capsulatus CA overexpression*		
CAH169 MCA1422 F	gtgatagagaaaagtgaaATGGAAGCTTTCGAGCGAATG	Amplify 645 bp MCA1422 ORF for overexpression construct
CAH170 MCA1422 R	cttcacaggtcaagcttCTATTCGGAGAAATCGACGTAC	
CAH171 MCA1665 F	gtgatagagaaaagtgaaGTGCAAGAGTCTGACAGC	Amplify 717 bp MCA1665 ORF for overexpression construct
CAH172 MCA1665 R	cttcacaggtcaagcttTCAATCGGTCACAGGCAAG	
CAH173 MCA2797 F	gtgatagagaaaagtgaaATGGCGATTCAGACCTAC	Amplify 543 bp MCA2797 ORF for overexpression construct
CAH174 MCA2797 R	cttcacaggtcaagcttCTAAGCGGCTCTGCC	
CAH175 MCA0910 F	gtgatagagaaaagtgaaATGTCACAGATTCTCAGCGAAGTACTG	Amplify 579 bp MCA0910 ORF for overexpression construct
CAH176 MCA0910 R	cttcacaggtcaagcttCTAAGCGGCTCTGCCCGC	
CAH177 MCA1080 F	gtgatagagaaaagtgaaATGGTACGGCGAACCATTTG	Amplify 795 bp MCA1080 ORF for overexpression construct
CAH178 MCA1080 R	cttcacaggtcaagcttTCACGGATCGACGTTCCTG	
CAH1426 MCA1080 R	TCACGGATCGACGTTCCTG	Pair with oCAH004 to linearize pCA2 and insert MCA1422 with native RBS
CAH1431 MCA1422 F	cgatccgtgaAACCTAAGAGCGAACTCATG	Pair with oCAH170 to amplify MCA1422 with native RBS
CAH003 pCAH01 R	TTCACTTTTCTCTATCACTGATAG	Linearize pCAH01 for CA overexpression construction
CAH004 pCAH01 F	AAGCTTGACCTGTGAAGTG	
CAH005 pCAH01 seq F	CCCGACACCATCGAATGGCCAGATG	Sequencing primers for overexpression constructs
CAH006 pCAH01 seq R	AGGGCGCGTGGAGATCCGT	
*M. capsulatus CA qRT-PCR*		
CAH350 MCA0910 RT F	TGTCACAGATTCTCAGCGAAG	qRT-PCR primers for MCA0910
CAH351 MCA0910 RT R	TCCATGCAGGTCAATATAGCG	
CAH352 MCA1080 RT F	ACCTTATCAACATCCACGAGC	qRT-PCR primers for MCA1080
CAH353 MCA1080 RT R	TCTGCTTGTTCTGATGGACG	
CAH344 MCA1442 RT F	AACAAATGGCTGAAACACGTC	qRT-PCR primers for MCA1442
CAH345 MCA1442 RT R	GTGGGCCAGATTGTATACCTG	
CAH346 MCA1665 RT F	CAGCAAAGGCAAGGATGAATG	qRT-PCR primers for MCA1665
CAH347 MCA1665 RT R	TCCTTGAATCCTTTGACGCC	
CAH348 MCA2797 RT F	CACTGAAGGACTCCGAACTC	qRT-PCR primers for MCA2797
CAH 349 MCA2797 RT R	CGCAAATACCGCTCCTTTAG	

^*a*^ Lowercase bases represent homology arms for isothermal assembly.

Overexpression plasmids and strains were created by amplifying each CA open reading frame from *M. capsulatus* Bath gDNA using the corresponding primers (oCAH169-oCAH178) and assembling with pCAH01 linearized *via* PCR with primers oCAH3/oCAH4 to create pCA1-pCA5, placing the genes under transcriptional control of the anhydrotetracycline (aTc)-inducible P*_tet_* promoter. To generate the pCA2/3 plasmid for co-expression of CA2 and CA3, the CA3 ORF with its native ribosome binding site was amplified from gDNA with primers oCAH1431/oCAH170 and assembled with pCA2 linearized with primers oCAH1424/oCAH4, generating a CA2-CA3 operon. Overexpression constructs were then transferred to *M. capsulatus* Bath *via* biparental mating. Antibiotic-resistant *M. capsulatus* transformants were screened *via* colony PCR using plasmid-specific primers oCAH5/oCAH6 and the resultant PCR amplicon sequence was verified *via* Sanger sequencing.

### CA alignment and phylogenetic analysis

The *M. capsulatus* CA protein sequences were aligned to respective homologous CA sequences with solved structures using Geneious Prime. Other CA homologs were identified in the NCBI database *via* BlastP using a standard BLOSUM62 matrix. Methanotroph CA homologs were identified in the NCBI database limiting the search to include Methylococcaceae, Methylothermaceae, Methylocystaceae, and Beijerinckiaceae methanotroph-containing families and excluding the *Methylococcus* genus. Distance trees of the top hits with query coverage ≥80% and E values ≤ 1e^−30^ were constructed with NCBI Blast Tree using a Fast Minimum Evolution method.

### CA activity assay

Wild-type *M. capsulatus* Bath cultivated in 10 mL liquid NMS to OD_600_ ~ 1.0 were pelleted at 4,080 × *g* for 10 minutes, and the supernatant was removed *via* aspiration. Cells were lysed using the B-PER bacterial protein extraction reagent (Thermo-Fisher) and total protein was quantified on a Nanodrop spectrophotometer using the Pierce 660_nm_ Protein Assay Reagent (Thermo-Fisher) compared to known BSA standards. Lysates were normalized to 10 mg/mL protein in 10 mM Tris HCl, and 100 µg of lysate was used to determine CA activity following the Abcam Carbonic Anhydrase Colorimetric Activity Assay Kit protocol (Abcam). Briefly, cell lysate and kit reagents were added to microtiter plates and allowed to incubate for 10 minutes at room temperature covered/protected from light and then production of nitrophenol was monitored at A_405nm_ every 30 seconds for 10 minutes using a BioTek Synergy Mx microplate reader. Nitrophenol was quantified *via* regression analysis compared to known nitrophenol standards which correlates to CA activity based on activity curves previously generated by the manufacturer. Acetazolamide (AZM) was added to a final concentration of 100 µM to inhibit CA activity in reactions where indicated.

### AZM treatment

Wild-type or AZM-adapted *M. capsulatus* Bath were grown in a liquid medium to OD_600_ ~1 and diluted to OD_600_ = 0.1 in fresh medium with or without AZM (Sigma-Aldrich). Cell death and/or growth was monitored *via* dilution plating and spectrophotometrically at OD_600_ using a Nanodrop spectrophotometer. % killing was calculated by comparing the colony forming units (CFUs) after treatment to CFUs at T = 0.

### CA transcription analysis *via* quantitative reverse-transcriptase PCR

CA transcription was determined in wild-type *M. capsulatus* cultivated in serum vials or a continuous gas bioreactor. Serum vial seed cultures were inoculated to OD_600_ = 0.1 in liquid NMS from plate biomass and cultivated for 24 h. Seed cultures were diluted in fresh medium to OD_600_ = 0.01 (serum vials) or OD_600_ = 0.1 (bioreactor). Serum vial cultivations were grown until the mid-log phase (OD_600_ = 0.5) for RNA extraction. To evaluate CA transcription in response to CO_2_ starvation, bioreactor cultures were cultivated with continuous 20% CH_4_/2% CO_2_ in the air for 3 h (CO_2_-replete) after which the gas composition was switched to 20% CH_4_ in the air (CO_2_-deplete) and cells were cultivated for an additional 3 h prior to RNA extraction. Cell cultures were mixed 5:1 with ice-cold 5% phenol/95% ethanol and incubated for 20 minutes on ice prior to RNA isolation using the High Pure RNA Isolation Kit with optional on-column DNase treatment (Roche). Total RNA (1 µg) was used to generate cDNA using the Superscript Vilo IV reverse transcriptase kit following the manufacturer’s protocol (Invitrogen). Primers used for qPCR are listed in Table 2. Reactions were prepared using Powertrack SYBR Green master mix (Applied Biosystems) using recommended cycling conditions and amplification detection with a QuantStudio 3 qPCR system. Expression of each CA determined by cycle threshold (Ct) was normalized to the housekeeping gene *rpoD* and relative expression between targets or conditions was calculated using the ∆∆Ct method.

### Gas chromatography and biomass yield calculations

Wild-type *M. capsulatus* and overexpression strains (pCA1-pCA5) were grown overnight in a liquid medium (OD_600_ ~0.1) with 50 µg/mL kanamycin and 0.2 µg/mL aTc for CA induction, where applicable. Seed cultures were sub-cultured to OD_600_ = 0.1 in fresh medium without antibiotics (to avoid any growth inhibition) and 0.2 µg/mL aTc for CA induction in serum vials, which were stoppered, sealed, and 20% CH_4_ added to the headspace *via* syringe. The CH_4_ and CO_2_ composition in the headspace was determined 10 min after gas addition and every 24 h during methanotroph cultivation using an SRI 8610 c gas chromatograph combined with an SRI S.S HayeSep D 6′ × 1/8″ column, SRI on-column injector, and TCD and FID detectors (SRI Instruments). Runs were 3 minutes in length, isocratic at 50°C. Gases were quantified as % composition by comparison to known standards. % gas was converted to weight using the specific gravities of CH_4_ (0.622 mg/mL) or CO_2_ (1.47 mg/mL) at 37°C. CH_4_ uptake and CO_2_ evolution rates per dry cell weight (DCW) were determined by converting OD_600_ to DCW using a formula empirically determined by comparing OD_600_ measurements to colony forming units during serum vial cultivation (OD_600_ = 1 is equivalent to 0.254 ± 0.0249 g DCW/L). The biomass yield/carbon conversion efficiency (CCE) at the late logarithmic growth phase (48 h) was calculated using the following equation: g DCW/(mmol CO_2_ evolved/mmol CH_4_ consumed).

### Competitive growth index

Wild-type *M. capsulatus* and antibiotic-resistant CA overexpression strains were equally mixed/subcultured to OD_600_ ~0.01 in NMS liquid medium and cultured for 48 h to late logarithmic phase. Serial dilutions of the cultures were replica plated on NMS and NMS containing 50 µg/mL kanamycin before and after cultivation, and CFUs were enumerated after 1 week of incubation. Competitive indices were calculated by determining the ratio of CA-overexpression strain CFUs to wild-type CFUs after cultivation (output ratio) divided by the CFU inoculum ratio (input ratio) using the following equation: output ratio [NMS Kn CFUs- (NMS CFUs - NMS Kn CFUs)] / input ratio [NMS Kn CFUs - (NMS CFUs - NMS Kn CFUs)].

### Statistical analysis

Statistical analysis was performed using GraphPad Prism 10 software. The data between the two groups were analyzed using unpaired *t*-tests. The determination of statistical significance between multiple comparisons was achieved using analysis of variance (ANOVA) followed by Dunnett’s multiple comparisons test. Data were considered statistically significant when *P* ≤ 0.05.

## RESULTS

### *M. capsulatus* Bath encodes five carbonic anhydrase isoforms

**Fig 1:**
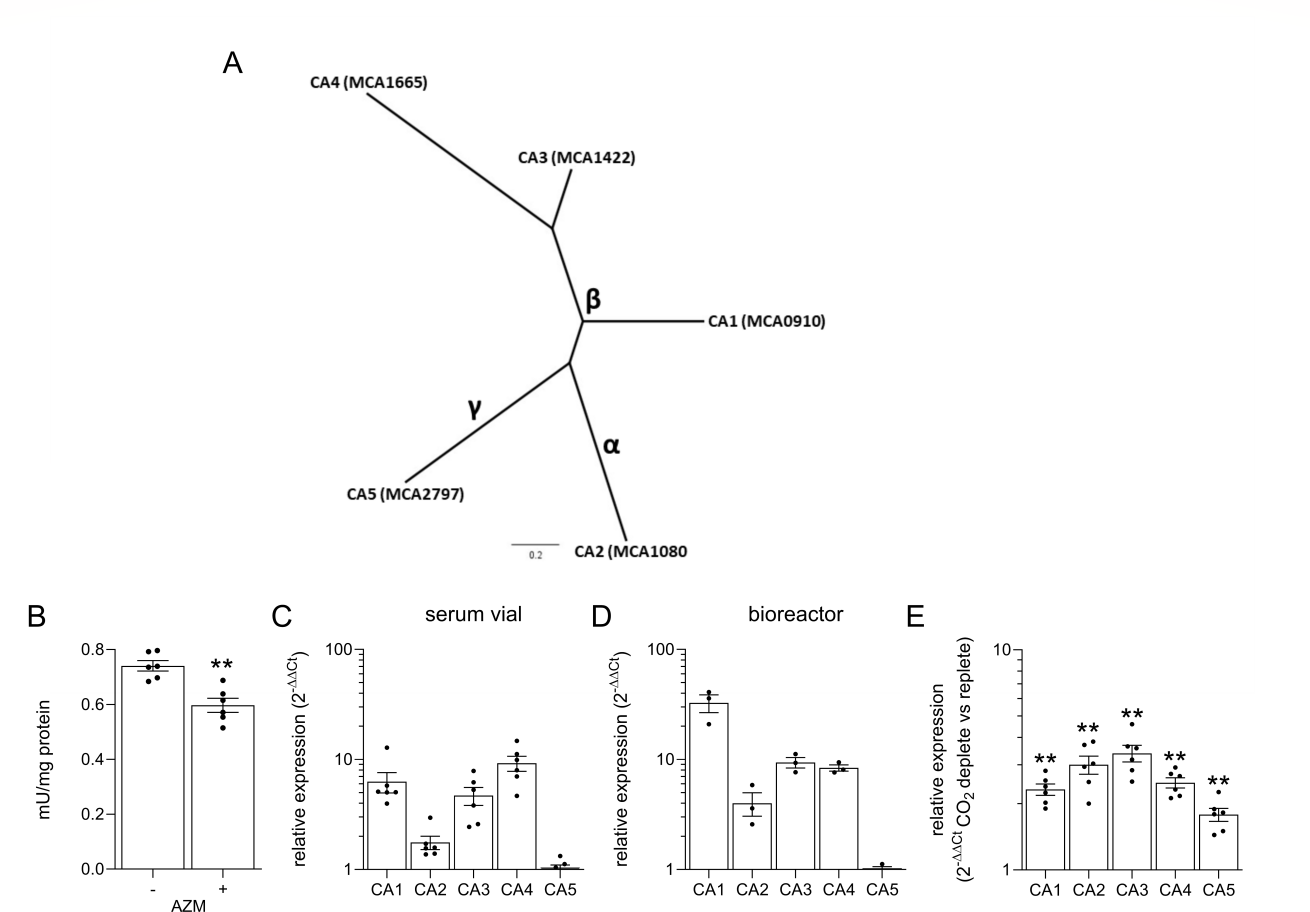
*M. capsulatus* expresses five CA isoforms. (**A**) Phylogenetic tree of the five annotated CAs encoded by the *M. capsulatus* Bath genome. Locus tags are indicated in parentheses. The scale bar represents substitutions per site. (**B**) CA activity was measured in whole-cell lysates in the presence (+) or absence (−) of the CA inhibitor acetazolamide (AZM). Relative transcription of the CA isoforms determined by qRT-PCR during *M. capsulatus* cultivation in (**C**) serum vials with 20% CH_4_ in air or (**D**) a gas reactor supplied with a continuous mixture of 20% CH_4_/2% CO_2_ in air. (**E**) Relative CA transcription a a continuous gas reactor 3 h after CO_2_ depletion from the gas mixture. Data in **B**–**E** represent the mean ± SEM from at least two independent experiments (*n* = 3–6). **P ≤ 0.01.

Five CA-encoding genes are annotated in the *M. capsulatus* Bath genome: MCA0910 (CA1), MCA1080 (CA2), MCA1442 (CA3), MCA1665 (CA4), and MCA2797 (CA5). The genes encode α-(CA2), γ-(CA5), and β-CAs (CA1, clade D; CA3, clade A, CA4, clade B) with limited amino acid sequence identity (Fig. 1A), but have well-conserved features to known CA families (Fig. S1 to S5) (27, 44–46). Phylogenetic analyses indicate that β-CA1, β-CA3, and γ-CA5 are highly conserved in methanotrophic bacteria, and the CA phylogenetic trees are congruent with 16s- and *pmoA*-based methanotroph classifications (8) (Fig. S6, S8, S10, S11, S13 and S15; supplemental table file S1 and S2). By contrast, α-CA2 and β-CA4 isoform orthologs share limited sequence identity (<30%) with other methanotroph CAs (Fig. S7, S9, S12, and S14; supplemental table file S2), while orthologs in non-methanotrophic bacteria with higher sequence identity (>50%) were identified, suggesting these isoforms may have been acquired *via* horizontal gene transfer. The phylogenetic analysis of the *M. capsulatus* α-CA2 identified orthologs in phylogenetically diverse phototrophic green non-sulfur bacteria, purple sulfur bacteria, and chemolithoautotrophic bacteria (Fig. S2 Fig. S7; File. S1). Furthermore, α-CA2, but not the other isoforms, contains a predicted Sec-specific signal sequence identified by SignalP (47), suggesting that this CA is secreted to the periplasm. CA activity can be detected in *M. capsulatus* whole-cell lysates (0.74 ± 0.05 mU/mg protein), which could be partially inhibited by the carbonic anhydrase inhibitor acetazolamide (AZM, Fig. 1B). The CA activity assay we used is based on the ability of CA to hydrolyze p-nitrophenol acetate. Thus, the limited (20%) decrease in CA activity we observed after AZM treatment may be explained by (i) most of the activity is due to non-CA esterases in the cell lysate or (ii) the majority of *M. capsulatus* CA activity is AZM-resistant.

Quantitative real-time PCR established that all CA isoforms were transcribed during bacterial cultivation in a serum vial with CH_4_ as the only supplied carbon source and in a bioreactor with continuous CH_4_ and CO_2_ supply (Fig. 1C and D). β-CA4 showed the highest relative expression in serum vials, which was 9.3-fold higher than the lowest expressed γ-CA5. Relative expression of β-CA1 was 3.6-fold higher (*P* < 0.05) in a bioreactor culture compared to a serum vial, but the expression of the other CA genes was comparable between these two growth conditions (Fig. 1C). The difference in expression between the highest (β-CA1) and lowest (γ-CA5) expressed CA genes was 32.7-fold in bioreactor-grown cells (Fig. 1D). To evaluate whether *M. capsulatus* Bath regulates CA expression in response to CO_2_ availability, we compared CA transcription in bioreactor-cultivated cells after the removal of CO_2_ from the gas stream (CO_2_-deplete) to those cultured with continuous 20% CH_4_ and 2% CO_2_ in air (CO_2_-replete) (Fig. 1E). In support of the role of the CAs in CO_2_ metabolism, transcription of all isoform genes was significantly increased within 3 h after CO_2_ starvation (CA1 2.6-fold; CA2 3.4-fold; CA3 3.8-fold; CA4 2.8-fold, and CA5 2.0-fold).

### The carbonic anhydrase inhibitor acetazolamide inhibits *M. capsulatus* growth

**Fig 2:**
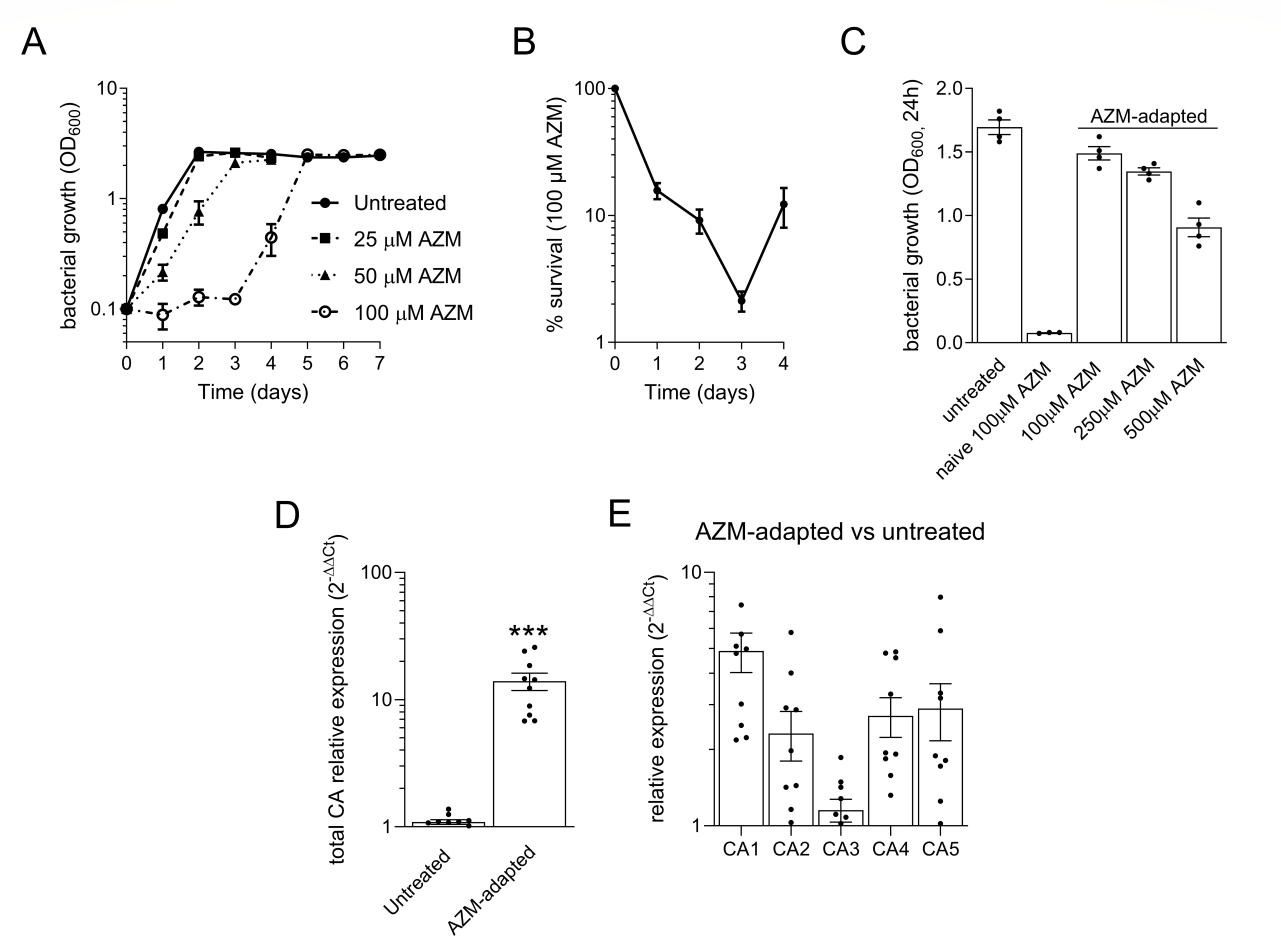
*M. capsulatus* is hypersusceptible to carbonic anhydrase inhibition. (**A**) Bacterial growth in liquid NMS medium with the acetazolamide (AZM) CA inhibitor compared to an untreated control. (**B**) Survival of *M. capsulatus* after exposure to 100 µM AZM. (**C**) Bacterial growth of AZM-adapted cells in the presence of AZM compared to naïve and untreated controls. Collective (**D**) or individual (**E**) CA transcription in 10 AZM-adapted transformants compared to wild-type controls was evaluated using qRT-PCR. The data represent the mean ± SEM from at least two independent experiments (*n* = 3–10). ***P ≤ 0.001.

The importance of CA activity for bacterial growth was initially tested by treating cells with increasing concentrations of AZM. AZM showed dose-dependent *M. capsulatus* growth inhibition with 100 µM AZM-treated cultures exhibiting a 3-day lag phase followed by growth with similar kinetics to untreated cells (open circles, Fig. 2A). Dilution plating of AZM-treated cells supported that the CA inhibitor is bactericidal, killing 98% of the treated cells within 72 h after exposure (Fig. 2B). However, a population of cells survived and began to expand after 96 h (Fig. 2B), which was not due to AZM degradation given that AZM maintains its growth inhibitory effect after abiotic incubation in NMS medium at 37°C for up to 10 days (data not shown). We did not rule out the possibility that AZM was biologically consumed or degraded under our experimental conditions. However, the hyper resistance of AZM-treated cells to AZM concentrations that completely inhibited the growth of naïve controls indicated that *M. capsulatus* adapts to overcome AZM-mediated toxicity (Fig. 2C). To test whether the CAs may be related to the AZM adaptation, we quantified CA transcription in 10 independent colonies from the AZM-adapted population and found that each strain showed increased overall CA expression ranging from 6.8- to 25.8-fold compared to collective wild-type CA expression levels (Fig. 2D), linking AZM resistance to increased CA expression. However, there was variation between which CA isoforms were induced, and to what extent, in the adapted strains preventing a direct correlation of AZM resistance to a particular CA isoform (Fig. 2E).

### Carbonic anhydrase mutants exhibit defective growth

**Fig 3:**
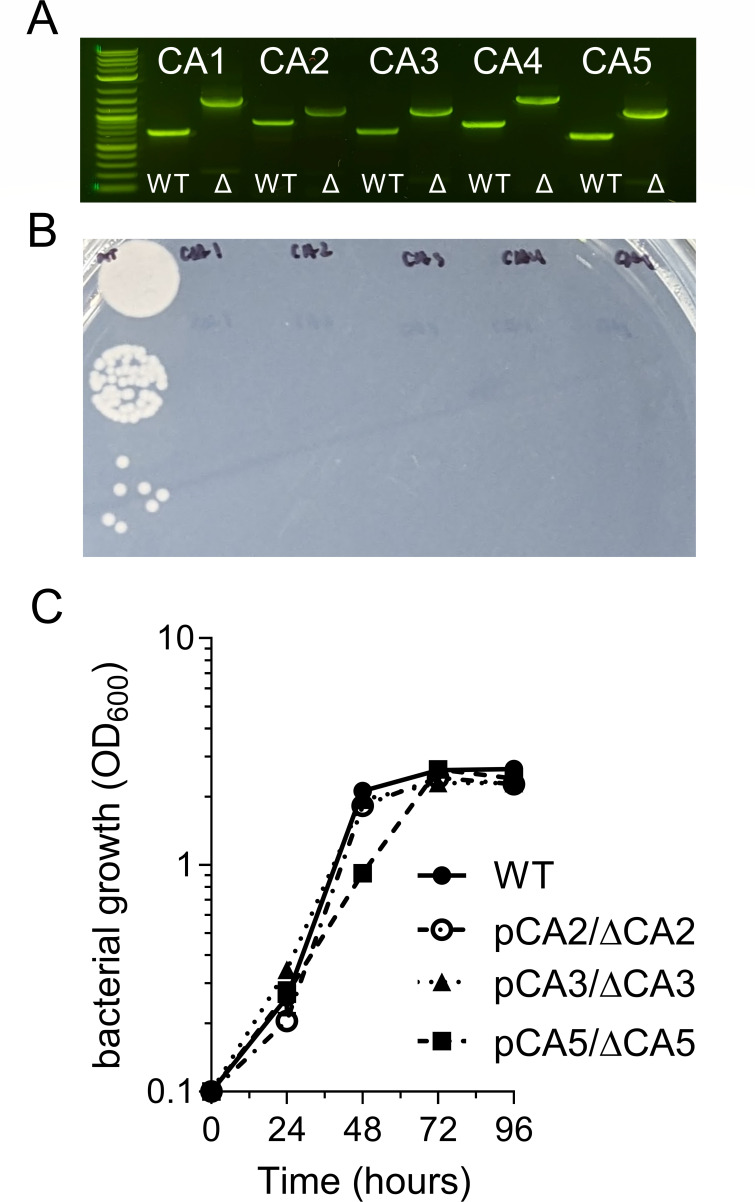
CA-deficient *M. capsulatus* exhibits defective growth. (**A**) Confirmation of individual CA knock-out strains *via* PCR. CA genes were replaced with antibiotic resistance cassettes *via* marker-exchange mutagenesis and knock-out (∆) strains were confirmed *via* PCR compared to WT. (**B**) Comparison of colony formation 1 week after incubation of a dilution plate of equivalent WT and CA knock-out strains (CA1-5). (**C**) Bacterial growth of wild-type and ∆CA2, ∆CA3, and ∆CA5 knock-out strains ectopically expressing a respective CA from a replicative plasmid. We were unable to complement the ∆CA1 and ∆CA4 strains. Data in **C** represent the mean ± SEM from two independent experiments (*n* = 4).

To assess the role of the individual CAs, we constructed five CA-deficient strains lacking each individual CA isoform *via* marker-exchange mutagenesis (ΔCA1::Kn^R^, ΔCA2::Gm^R^, ΔCA3::Gm^R^, ΔCA4::Kn^R^, and ΔCA5::Gm^R^). Antibiotic-resistant transformants were isolated on solid NMS medium and individual CA knock-out strains were confirmed *via* PCR (Fig. 3A). These CA knock-out transformants appeared after 4–8 weeks of incubation compared to isogenic wild-type *M. capsulatus* Bath that formed visible colonies within 1–2 weeks, underscoring that each CA isoform has a critical, non-redundant role in *M. capsulatus* metabolism and physiology. The extreme growth defects of the CA knock-out strains precluded reasonable growth comparison in liquid NMS medium; thus, we spotted dilution series of plate-derived wild-type and CA KO *M. capsulatus* strains on solid medium (Fig. 3B). After 10 days of incubation, wild-type colonies were readily visible, but the CA KO strains had no observable growth, even in the undiluted spots (Fig. 3B). We introduced a plasmid with tetracycline promoter (P_tet_)/aTc-inducible expression of a CA into its respective CA KO background strain to confirm that the observed growth defects were attributed to the removal of the CA and not unintended genetic defects. Growth defects were complemented by ectopic expression of CA2, CA3, and CA5 in strains pCA2 ΔCA2::Gm^R^, pCA3 ΔCA3::Gm^R^, and pCA5 ΔCA5::Gm^R^, respectively (Fig. 3C). However, we were unable to recover growth of ΔCA1::Kn^R^ and ΔCA4::Kn^R^ strains, potentially due to dysregulated expression of CA1 or CA4 from the pCAH01 P*_tet_* promoter or off-target effects resulting from removal of the CA gene. An 86-bp intergenic region exists between CA1 and the downstream gene and CA4 appears to be the first gene in an operon with a downstream gene (the CA4 stop codon and downstream MCA1664 start codon are separated by 11 bp). However, the putative ribosomal binding site at the 3′ ends of the CA4 open reading frame was maintained in the ΔCA4::Kn^R^ strain to ensure translation of the downstream gene transcript, but it is possible that removal of the CA1 or CA4 sequences could disrupt regulatory elements that control the expression of the downstream genes resulting in the observed growth defects. Collectively, the AZM toxicity toward *M. capsulatus* and the significant growth defects of the CA knock-out strains underscore that CA activity is critical for optimal *M. capsulatus* growth.

### Carbonic anhydrase overexpression improves *M. capsulatus* growth and increases carbon conversion efficiency

**Fig 4:**
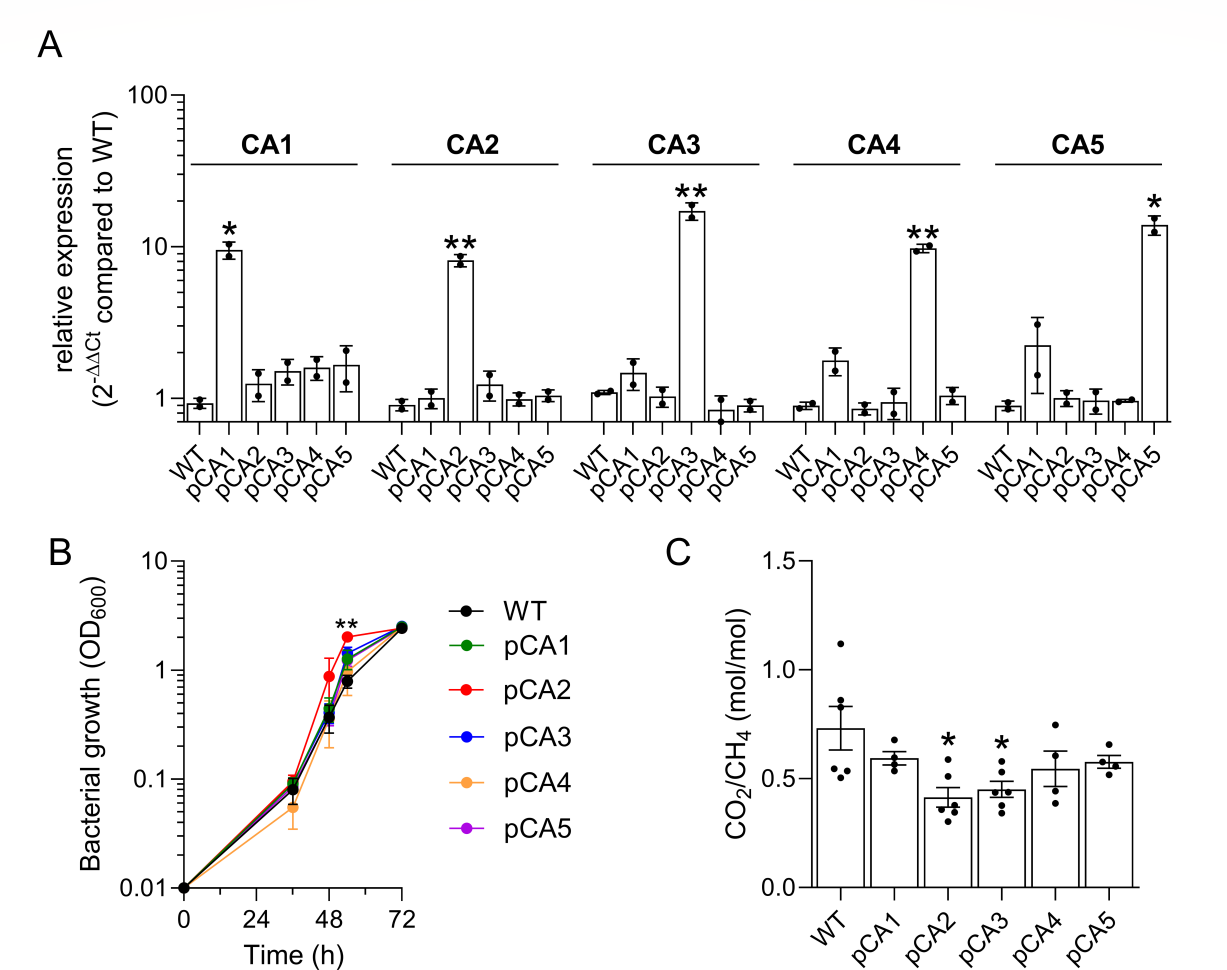
CA overexpression boosts *M. capsulatus* growth. (**A**) CA isoforms were overexpressed from the pCAH01 broad-host-range expression vector. Individual CA transcription in overexpression strains was compared to WT *via* qRT-PCR. (**B**) Bacterial growth of WT and CA overexpressing strains in serum vials supplied with 20% CH_4_ in air. (**C**) The ratio of CO_2_ evolved to CH_4_ consumed by WT and CA overexpression strains to reach mid-logarithmic growth (OD_600_ ~0.5) determined by gas chromatography. The data represent the mean ± SD (**A**) or SEM (**B and C**) from at least two independent experiments (*n* = 2–6). *P ≤ 0.05. **P ≤ 0.01.

**Fig 5:**
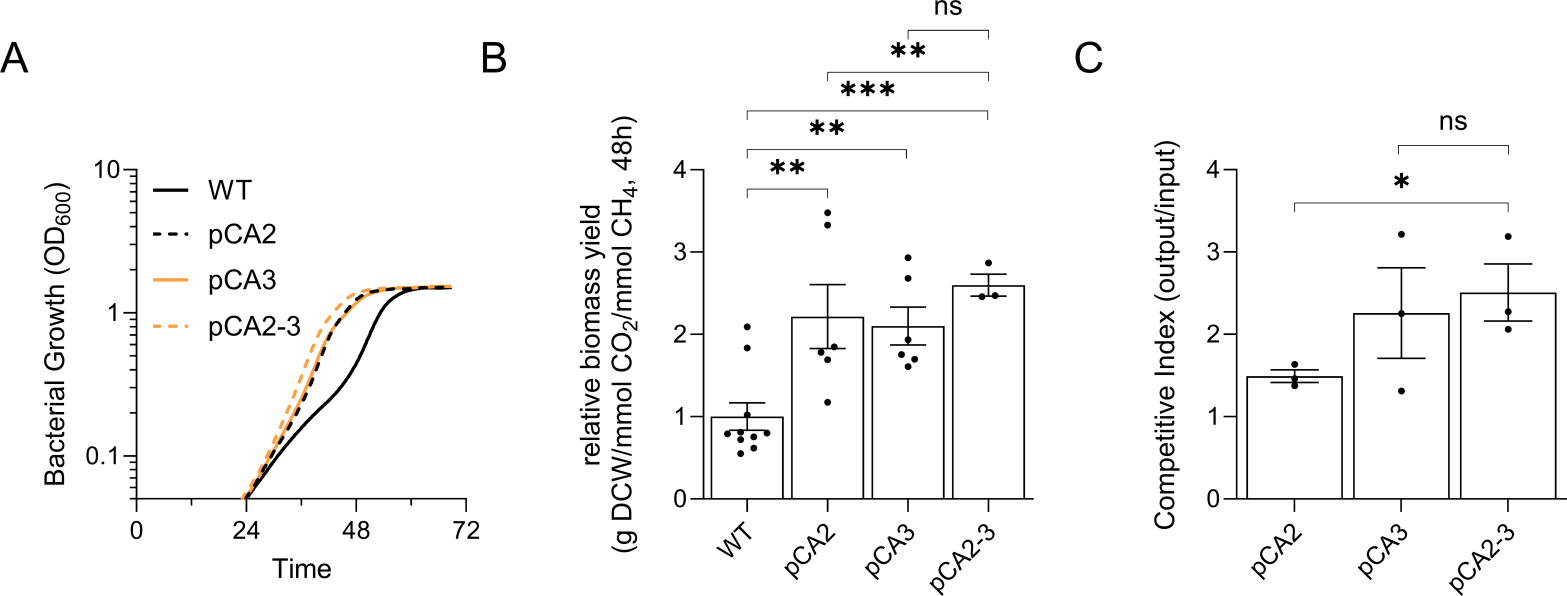
CA overexpression improves *Methylococcus* carbon conversion efficiency. (**A**) High-resolution cell growth quantification (CGQ) and (**B**) biomass yield calculated using the CO_2_ evolved/CH_4_ consumed ratio of WT and strains overexpressing CA2 (pCA2) or CA3 (pCA3) alone or in combination (pCA2-3). (**C**) Mixed cultures consisting of WT *M. capsulatus* and pCA2, pCA3, or pCA2-3 were cultivated for 48 h to evaluate bacterial fitness. The ratio of engineered strain (pCAx) to WT after 48 h was compared to the ratio of the inoculum by replica dilution plating on NMS solid medium with (pCAx only) or without kanamycin (WT + pCAx). The limit of detection of a CGQ sensor is OD_600_ ~0.5, which cultures reached at ~24 h post-inoculation. The data represent the mean of two independent growth experiments (**A**) or the mean ± SEM (**B and C**) from two independent experiments (*n* = 3–6). *P ≤ 0.05, **P ≤ 0.01, ***P ≤ 0.001.

The P_tet_-inducible CA expression plasmids developed for complementation of the KO strains were also transferred to wild-type *M. capsulatus* to analyze the effect of each isoform on *M. capsulatus* growth and C1 gas consumption/evolution. Overexpression of each isoform was confirmed *via* qRTPCR, with each isoform being induced ~10 fold over baseline when transcribed from the P_tet_ promoter (Fig. 4A). Growth analyses of the CA overexpression strains in serum vials with CH_4_ supplied as the only carbon source showed that increased expression of CA2 (pCA2) or CA3 (pCA3) isoforms enhanced growth compared to wild-type cultures with basal-level expression (Fig. 4B; Fig. S16). The growth enhancement of pCA2 and pCA3 was isolated to the early lag phase of growth since all CA overexpression strains had similar specific growth rates as wild-type *M. capsulatus*, ranging from 0.094 to 0.112 h^−1^ during logarithmic growth (Fig. S16A and B). Also, the CA overexpression strains entered the stationary phase at similar OD as wild-type *M. capsulatus* and accumulated comparable biomass over the duration of cultivation (Fig. 4B; Fig. S16A). Since the positive effect of CA overexpression on growth was attributed to lag phase, we evaluated gas consumption/evolution dynamics within the first 48 h of cultivation, which is the time required for cells to reach mid-logarithmic growth/metabolic steady state under our growth conditions (Table 3). *M. capsulatus* and the pCAx strains consumed between 106 and 133 mmol CH_4_/g DW with no statistical difference between the overexpression strains and wild-type cultures (Table 3; Fig. S17A). Wild-type *M. capsulatus* evolved 76 ± 8 mmol/g DW CO_2_ within 48 h, which is consistent with prior gas consumption/evolution measurements reported by this methanotroph (9). However, CA overexpression strain cultures showed an 8–21 mmol CO_2_/g DW^−1^ decrease in gas evolution, with pCA2 and pCA3 evolving 55 ± 7 and 56 ± 3 mmol CO_2_/g DW^−1^, respectively (Table 3; Fig. S17B). As such, pCA2 and pCA3 evolved less CO_2_ per CH_4_ consumed (0.41 ± 0.05 and 0.45 ± 0.04 mol/mol, respectively) compared to wild-type (0.73 ± 0.10 mol/mol) (Fig. 4C; Table 3). We did not measure significant differences in biomass yield from CH_4_ between any of the pCAx overexpression strains and wild-type *M. capsulatus*, indicating that the amount of formaldehyde condensation with ribulose monophosphate as part of the RuMP pathway was unaltered by CA overexpression (Table 3). However, overexpression of CA1, CA2, CA3, and CA5 variants significantly improved carbon conversion to biomass efficiency (CCE) when the CO_2_ evolved/CH_4_ consumed ratio was considered as the overall carbon assimilated to biomass (Table 3). The pCA2 and pCA3 strains showed the best overall CCE (pCA2, 3.25 ± 0.57 mg DW/mol CO_2_/mol CH_4_; pCA3, 3.09 ± 0.34 mg DW/mol CO_2_/mol CH_4_) which correlated to 2.2- and 2.1-fold improvement in biomass yield compared to wild-type (1.47 ± 0.25 mg DW/mol CO_2_/mol CH_4_), respectively (Table 3), consistent with the boosted growth observed in these strains (Fig. 5A). Collectively, these results show that wild-type *M. capsulatus* and CA overexpression strains consumed comparable amounts of CH_4_ and had similar biomass yield directly from CH_4_, but CA2 and CA3 overexpression decreased CO_2_ accumulation in the serum vial headspace (Table 3). The decrease in CO_2_ was not due to a decrease in complete CH_4_ oxidation to CO_2_ with associated higher formaldehyde flux to biomass but rather is attributed to increased CO_2_ re-uptake, which improved biomass yield of the CA overexpression strains (Fig. 5).

**TABLE 3:** CA overexpression strain culture metrics (48 h, logarithmic growth phase)^*a*^

Strain	CH_4_ consumed (mmol/g DW)	CO_2_ evolved (mmol/g DW)	CO_2_/CH_4_(mol/mol)	Biomass yield (g/g CH_4_)	Biomass yield (mg DW/mol CO_2_/mol CH_4_)
WT	114 ± 10	76 ± 8	0.73 ± 0.10	0.59 ± 0.05	1.47 ± 0.25
pCA1	106 ± 6	62 ± 2	0.59 ± 0.03	0.60 ± 0.03	*2.85 ± 0.42
pCA2	133 ± 15	55 ± 7	**0.41 ± 0.05	0.51 ± 0.08	**3.25 ± 0.57
pCA3	127 ± 6	56 ± 3	**0.45 ± 0.04	0.50 ± 0.02	**3.09 ± 0.34
pCA4	131 ± 14	68 ± 5	0.55 ± 0.08	0.49 ± 0.05	1.57 ± 0.17
pCA5	112 ± 17	64 ± 9	0.58 ± 0.03	0.60 ± 0.10	*2.64 ± 0.35
pCA2-3	140 ± 8	57 ± 3	*0.41 ± 0.05	0.45 ± 0.03	***3.82 ± 0.34

^*a*^ Data represent the mean ± standard error of 3–10 biological replicates from at least two independent experiments. *P ≤ 0.05; **P ≤ 0.01; ***P ≤ 0.001 compared to WT based on a two-tailed student’s *t*-test.

We next evaluated the effect of CA2 and CA3 co-overexpression on *M. capsulatus* growth and gas consumption/evolution dynamics. High-resolution cell growth quantification supported that overexpression of both CA2 and CA3 improved growth compared to overexpression of either variant individually, with the pCA2-3 strain exhibiting a 14-h decreased lag phase and reaching logarithmic growth earlier than wild type (Fig. 5A). Like the individual CA overexpression strains, pCA2-3 did not accumulate more biomass/reach higher OD than wild type. The pCA2-3 strain’s improved productivity was attributed to a 2.6-fold improvement in CCE (3.82 ± 0.34 mg DW/mol CO_2_/mol CH_4_) over the wild type (Fig. 5B; Table 3), which was also significantly higher than the CCE improvements observed *via* overexpression of CA2 or CA3 individually. To further validate these observed CCE improvements, we performed a competition assay to compare the fitness of the pCA2, pCA3, or pCA2-3 engineered strains to wild type in a mixed culture with the hypothesis that the overexpression strains would outperform wild-type *M. capsulatus* due to increased CO_2_ uptake. Indeed, pCA2, pCA3, and pCA2-3 outperformed wild-type 1.49-, 2.25-, and 2.51-fold, respectively (Fig. 5C), which are fitness enhancements consistent with the growth enhancements and CCE improvements measured in axenic cultures (Fig. 5B). These data highlight that CA overexpression is a viable strategy to improve CCE in a RubisCO-encoding methanotrophic biocatalyst. Furthermore, the pCA2-3 strain represents an engineered biocatalyst with enhanced biomass productivity that could improve the techno-economics of *M. capsulatus*-based single-cell protein production.

## DISCUSSION

Methanotrophic bacteria play vital roles in biogeochemical cycling across the globe and mitigate climate change by direct capture of the greenhouse gas CH_4_. *M. capsulatus* and other Type Ib Gammaproteobacterial methanotrophs encode RubisCO which mediates CO_2_ assimilation essential for their growth, thereby limiting the overall CO_2_ evolution from biological CH_4_ oxidation. In this study, we have provided several lines of evidence that CAs have vital roles in the *M. capsulatus* inorganic metabolism needed for the growth of this obligate methanotroph. Our results show that increasing methanotroph CA levels improve biomass productivity while paradoxically decreasing CO_2_ evolution, improving overall CCE to biomass. Thus, the biocatalysts with enhanced CCE and the insights developed here can be leveraged for improving CH_4_ bioconversion processes and climate change mitigation.

*M. capsulatus* expresses five CA isoforms with phylogenetic relationships that provide insight into their possible function(s) in general methanotroph metabolism and/or the specialized dual CH_4_ and CO_2_ metabolism occurring in *M. capsulatus* that express RubisCO. The *M. capsulatus* β-CA1, β-CA3, β-CA4, and γ-CA5 isoforms have orthologs in Gamma- and Alphaproteobacterial methanotrophs with significant (>30%) sequence identified (Fig. S6 to S15; supplemental table files S1 and S2). The presence of these orthologs in the Gammaproteobacterial methanotroph *Methylotuvimicrobium alcaliphilum* 20Z that does not require CO_2_ for growth (18) suggests that these CAs may perform more generalized functions or have evolved unique functions in *M. capsulatus*. By contrast, *M. capsulatus* α-CA2 orthologs are not common in methanotrophs but are found in an array of chemolithoautotrophic and phototrophic bacteria, highlighting a strong relationship between this CA isoform with autotrophic inorganic carbon metabolism. Many *M. capsulatus* α-CA2 orthologs are found in facultative anaerobic phototrophs, including *Rhodospirillum* and *Ectothiorhodospira* (Fig. S7; File. S1). Notably, the *M. capsulatus* RubisCO large subunit’s closest non-methanotroph ortholog is found in *Ectothiorhodospira mobilis* (92% sequence identity) (48), so perhaps several interspecies genetic exchanges have occurred between *Methylococcus* and chemoautotrophic bacteria, playing a part in the development of the *Methylococcus* dual C1 metabolism.

The five CA genes are appreciably expressed, and our data indicate that CA transcription can be induced in response to a decrease in CO_2_ availability (Fig. 1). Furthermore, bacterial treatment with the CA inhibitor, AZM, elicited an adaptive response resulting in an AZM-resistant phenotype that, at least partially, is attributed to CA overexpression (Fig. 2). The AZM-resistant phenotype is maintained in the absence of AZM selective pressure, pointing to genetic mutation(s) as a mechanism underlying the phenotype. AZM and other sulfonamide CA inhibitors are currently being considered as antibacterial therapeutics for the treatment of antibiotic-resistant bacterial infections (49). Our data show bacteria can develop resistance to AZM; thus, the *M. capsulatus* AZM adaptation may provide insight into pathogenic bacterial resistance strategies. Whole-genome sequencing of the AZM-resistant strains may identify mutations leading to dysregulation of CA expression or other adaptive mechanisms. For applied methanotroph biotechnologies, the identified mutations/genes would represent putative metabolic engineering targets to further improve CO_2_ uptake and conversion.

We hypothesized that the *M. capsulatus* cytoplasmic CAs (e.g., β-CA1, β-CA3, β-CA4, and γ-CA5) would complement the loss of other isoforms, so we were surprised to find that each CA KO strain had a substantial growth defect compared to wild-type (Fig. 3). These data highlight that each CA plays a unique, critical role in *M. capsulatus* metabolism and physiology, but the underlying mechanisms remain undetermined. CO_2_ and HCO_3_^−^ are required for several biochemical reactions; thus, some *M. capsulatus* CAs may play distinct roles in maintaining the intracellular CO_2_/HCO_3_^−^ balance for biosynthetic processes or other physiological functions rather than capturing external CO_2_. Each CA isoform could have unique binding partners or localization in *Methylococcus*, which could also explain a lack of redundancy. The *M. capsulatus* α-CA2 is the only secreted CA, and based on the physiological role of α-CAs in other bacteria, likely converts CO_2_ to HCO_3_ in the periplasmic space (28, 50). The *M. capsulatus* gene encoding β-CA3 is in an operon with a gene annotated to encode a SulP family inorganic anion transporter and members of this protein family have been demonstrated to transport HCO_3_^−^ in cyanobacteria (30, 51, 52). In addition, the β-CA4 gene is in an operon with a downstream gene (MCA1664) annotated as a xanthine/uracil permease, which is thought to be the ancestral protein family of the SulP bicarbonate transporters (53). The *M. capsulatus* β-CA3 and β-CA4 genomic context implies that they may coordinate with a SulP transporter to either import or export HCO_3_^−^ across the periplasmic membrane. Replacement of the CA4 locus with an antibiotic resistance cassette may have negatively affected the expression of the downstream putative transporter, which may explain our inability to complement the CA4 knock-out strain.

**Fig 6:**
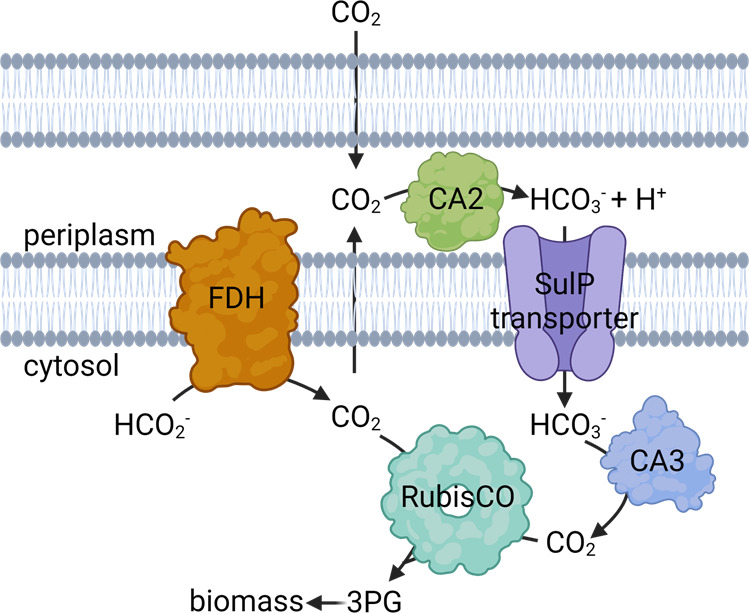
Working model for CO_2_ assimilation in *M. capsulatus*. CO_2_ is rapidly generated from the complete oxidation of CH_4_ in methanotrophs. Under low external CO_2_ partial pressures, CO_2_ produced intracellularly *via* enzymatic decarboxylation reactions or formate (HCO_2_^−^) dehydrogenase (FDH)-mediated oxidation of HCO_2_^−^ can rapidly diffuse from the cell. The α-CA2 located in the periplasmic space converts CO_2_ to HCO_3_^−^, which is imported by inorganic HCO_3_^−^/sulfate permease (SulP) family transporters. Intracellular CAs, including the β-CA3, catalyze HCO_3_^−^ dehydration to produce CO_2_ for assimilation by the ribulose-1,5-bisphosphate carboxylase/oxygenase (RubisCO). 3-Phosphglycerate (3 PG) generated by RubisCO enters an overlapping ribulose monophosphate/Calvin cycle to generate the C5 phosphosugars required for CH_4_ and CO_2_ assimilation and biomass production. Created with BioRender.com.

Notably, overexpression of CA2 or CA3 significantly increased CO_2_ assimilation compared to wild-type *M. capsulatus* (Fig. 4). The pCA2 and pCA3 strains do not have increased overall biomass accumulation compared to wild type, so the improved CCE phenotype seems to primarily be isolated to the lag phase. We speculate that after inoculation and introduction of CH_4_ only into the serum vial headspace, *M. capsulatus* completely oxidizes a significant percentage of CH_4_ to CO_2_ rather than biomass until it reaches a minimum partial pressure, at which time more CH_4_ is directed to biomass. Unfortunately, we do not yet have the resolution to define the exact CO_2_ partial pressure required for *M. capsulatus* to initially divide. CA overexpression may decrease the CO_2_ partial pressure required to initiate growth or could enable CO_2_ capture prior to release into the headspace. Although the mechanism is yet to be identified, CA overexpressing cultures evolve less CO_2_ per CH_4_ oxidized and the CO_2_ likely is converted to biomass since these strains exhibit improved productivity (i.e., faster biomass accumulation). We observed an additive growth enhancement with improved CO_2_ assimilation when the α-CA2 and β-CA3 were co-overexpressed (Fig. 4 and 5). Given that the β-CA3 is co-expressed with a bicarbonate transporter and the α-CA2 is secreted, these data lead us to a model wherein the α-CA2 converts CO_2_ to bicarbonate in the periplasm, which is imported across the periplasmic membrane by the SulP family transporter and converted to CO_2_ in the cytoplasm by the β-CA3, increasing RubisCO substrate availability (Fig. 6). This speculative carbon concentration mechanism needs to be experimentally tested but is a possible explanation for the phenotypes observed here.

Technoeconomic analyses have identified CCE and productivity as primary cost drivers in CH_4_ bioconversion processes using methanotrophic bacteria (54). A twofold improvement in CCE in natural gas to lactate conversion bioprocess can decrease the lactate minimal selling price ($/kg) by 34%, which is primarily due to an overall decrease in capital expenditures (54). The engineered strain developed here (pCA2-3) has a 2.5-fold increase in CCE (Fig. 5). As such, using this strain to convert CH_4_-rich natural gas or anaerobic digestion-derived biogas to valuable products (e.g., lactate) or microbial biomass/single-cell protein would immediately reduce production costs. Further strain improvements to enhance CO_2_ assimilation/CCE, increase product titer and productivity, and bioprocess optimization could lead to dramatic cost reductions to facilitate the commercialization of CH_4_ bioconversion processes.

Methanotroph-dependent conversion of CH_4_ represents an approach to capture GHGs and mitigate climate change. Here, we have advanced our understanding of the dual CH_4_ and CO_2_ metabolism occurring in the methanotroph, *Methylococcus capsulatus*, a methanotroph currently used industrially to produce single-cell protein. These and future insights into the *M. capsulatus* metabolism and physiology will guide strain improvement strategies required to realize the effective use of these biocatalysts in biotechnology applications.

## SUPPLEMENTAL MATERIAL

Supplemental figures msphere.00496-24-s0001.pdf Figures S1 to S17.

Tables S1 to S5 msphere.00496-24-s0002.xlsx Blastp results tables.

Tables S6 to S10 msphere.00496-24-s0003.xlsx Blastp methanotroph results tables.

ASM does not own the copyrights to Supplemental Material that may be linked to, or accessed through, an article. The authors have granted ASM a non-exclusive, world-wide license to publish the Supplemental Material files. Please contact the corresponding author directly for reuse.
