# Supplementary material for: Overexpression of native carbonic anhydrases increases carbon conversion efficiency in the methanotrophic biocatalyst Methylococcus capsulatus Bath
Source: mSphere. 2024 Aug 27;9(9):e00496-24. doi: 10.1128/msphere.00496-24 (PMC11423575; doi:10.1128/msphere.00496-24)
Supplement: Supplemental figures — Figures S1 to S17. [file msphere.00496-24-s0001.pdf]

Overexpression of native carbonic anhydrases increases carbon conversion efficiency in the methanotrophic biocatalyst *Methylococcus capsulatus* Bath.

Spencer A. Lee, Jessica M. Henard, Robyn A. C. Alba, Chance A. Benedict, Tyler A. Mayes, and Calvin A. Henard*

Supplemental Figures

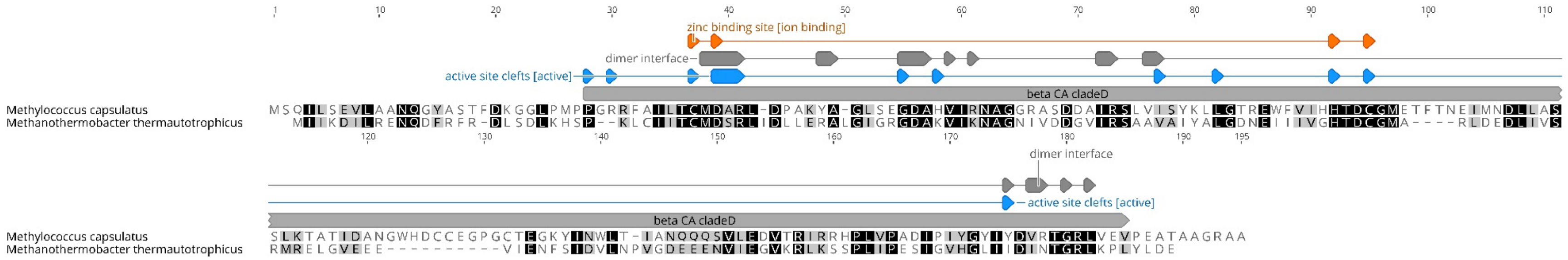

Figure S1. Protein alignment of the *M. capsulatus* Bath βCA1 (MCA0910) with the methanogen *Methanothermobacter thermautotrophicus* homolog (PDB ID: 1G5C). The conserved protein domain family (light grey), putative zinc binding sites (orange), dimer interface (dark grey), and active site clefts (blue) are indicated.

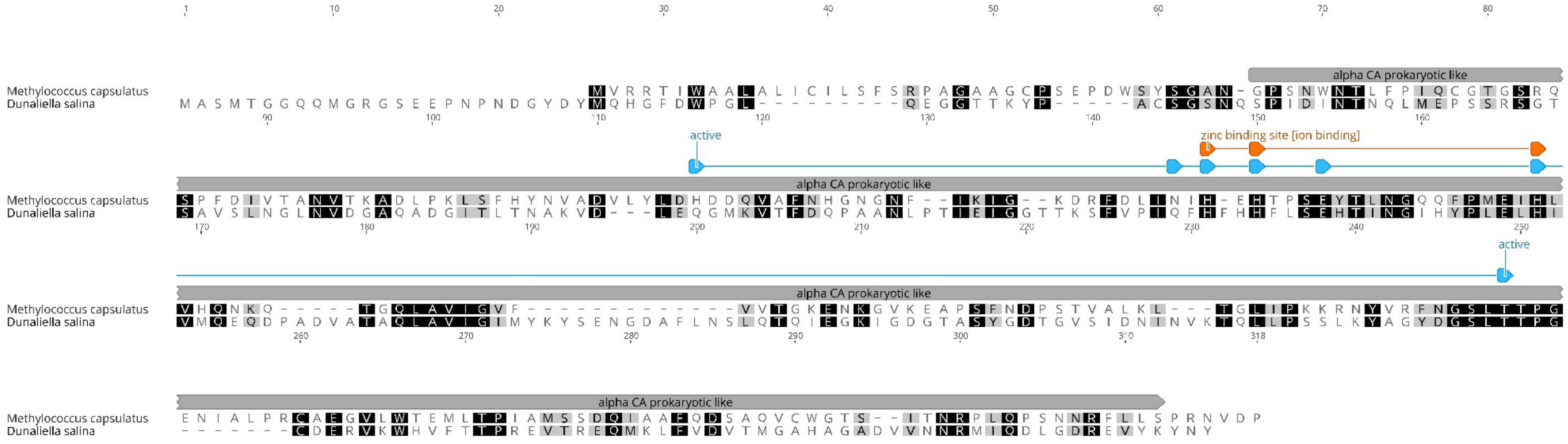

Figure S2. Protein alignment of the *M. capsulatus* Bath αCA2 (MCA1080) with the green microalga *Dunaliella salina* homolog (PDB ID: 1Y7W). The conserved protein domain family (grey), putative zinc binding sites (orange) and active site clefts (blue) are indicated.

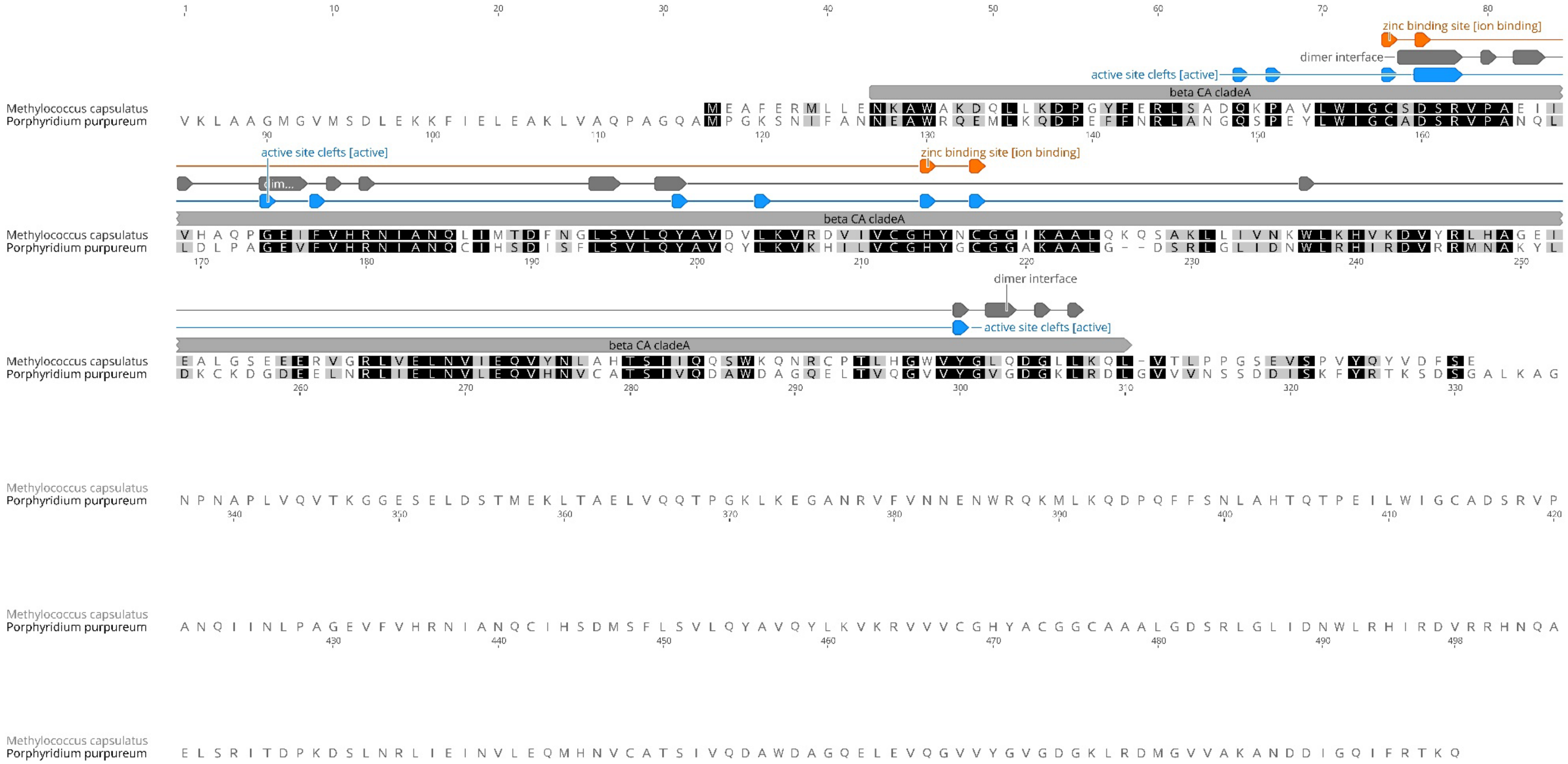

Figure S3. Protein alignment of the *M. capsulatus* Bath βCA3 (MCA1442) with the red microalga *Porphyridium purpureum* homolog (PDB ID: 1DDZ). The conserved protein domain family (light grey), putative zinc binding sites (orange), dimer interface (dark grey), and active site clefts (blue) are indicated.

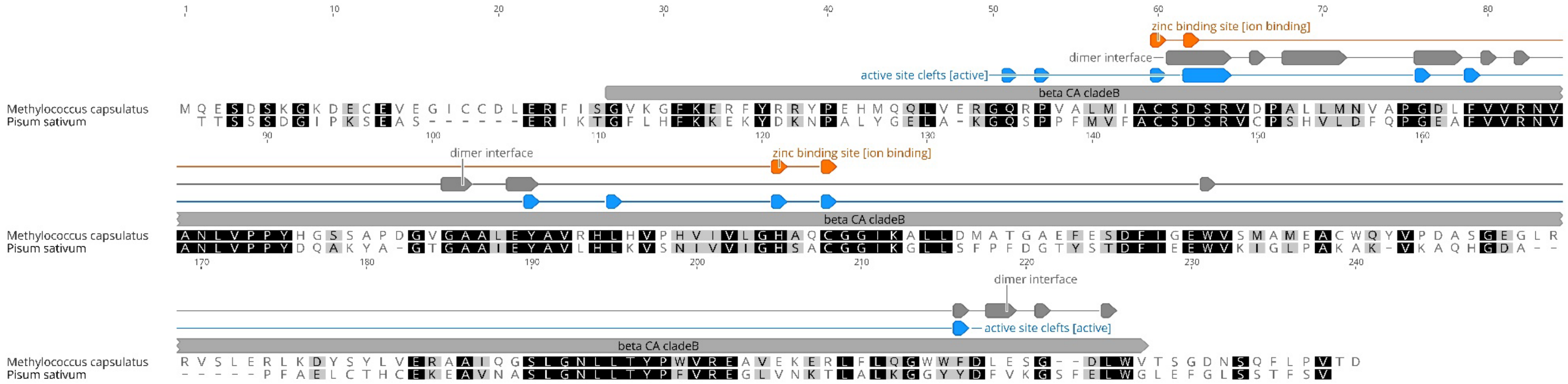

Figure S4. Protein alignment of the *M. capsulatus* Bath βCA4 (MCA1665) with the garden pea *Pisum sativum* homolog (PDB ID: 1EKJ). The conserved protein domain family (light grey), putative zinc binding sites (orange), dimer interface (dark grey), and active site clefts (blue) are indicated.

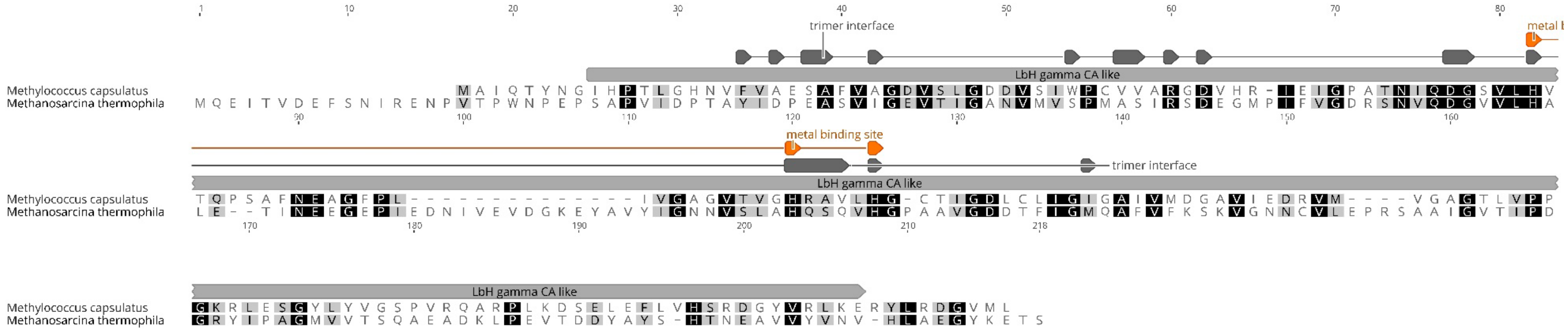

Figure S5. Protein alignment of the *M. capsulatus* Bath γCA5 (MCA2797) with the methanogen *Methanosarcina thermophila* homolog (PDB ID: 1THJ). The conserved protein domain family (light grey), putative zinc binding sites (orange) and trimer interface (dark grey) are indicated.

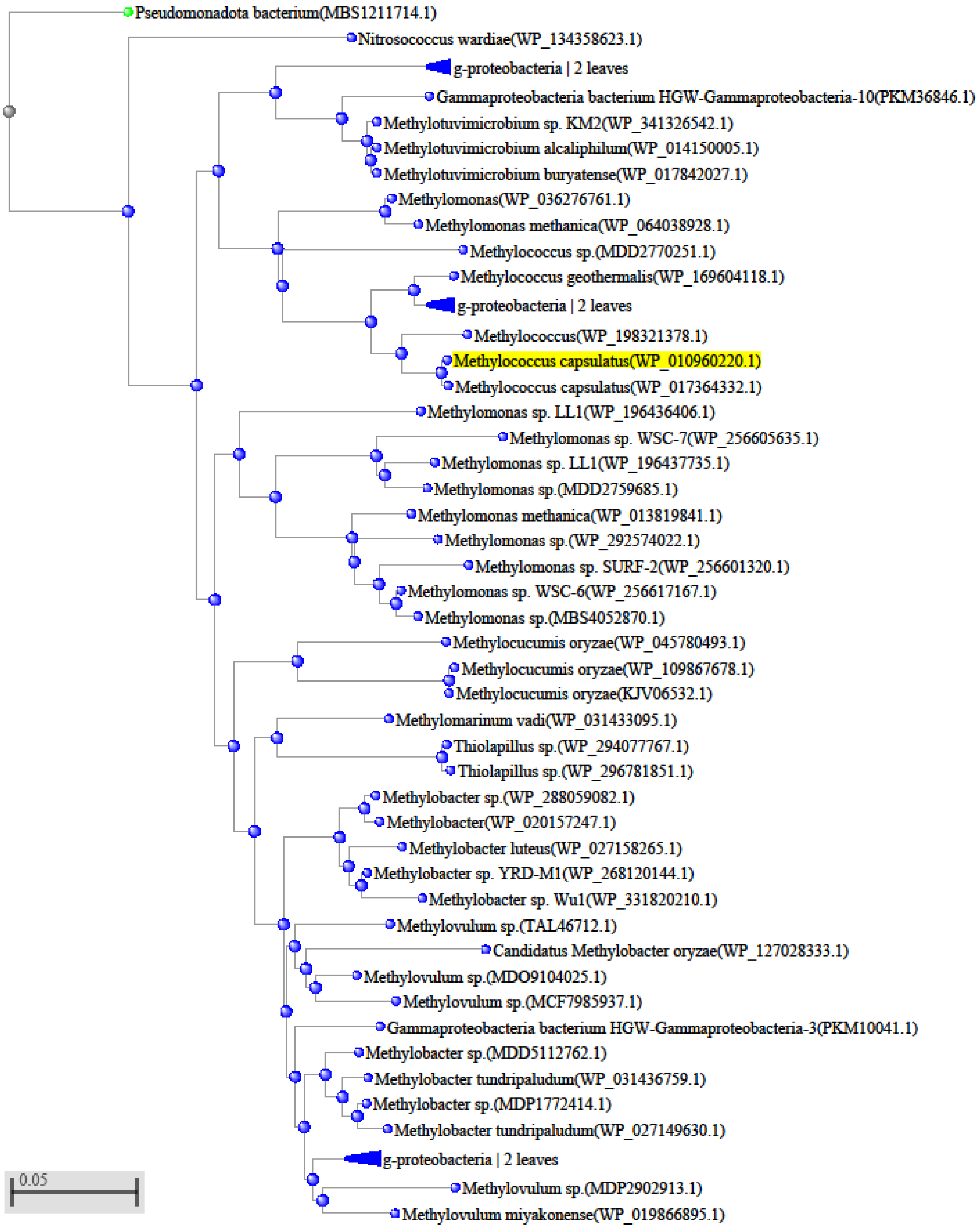

Figure S6. Cladogram of the *M. capsulatus* Bath CA1 (MCA0910) top 50 orthologs identified by Blastp. The *M. capsulatus* Bath query sequence is highlighted, Protein accession numbers are indicated in parentheses. *Gammaproteobacteria* (dark blue nodes). Scale represents substitutions per site.

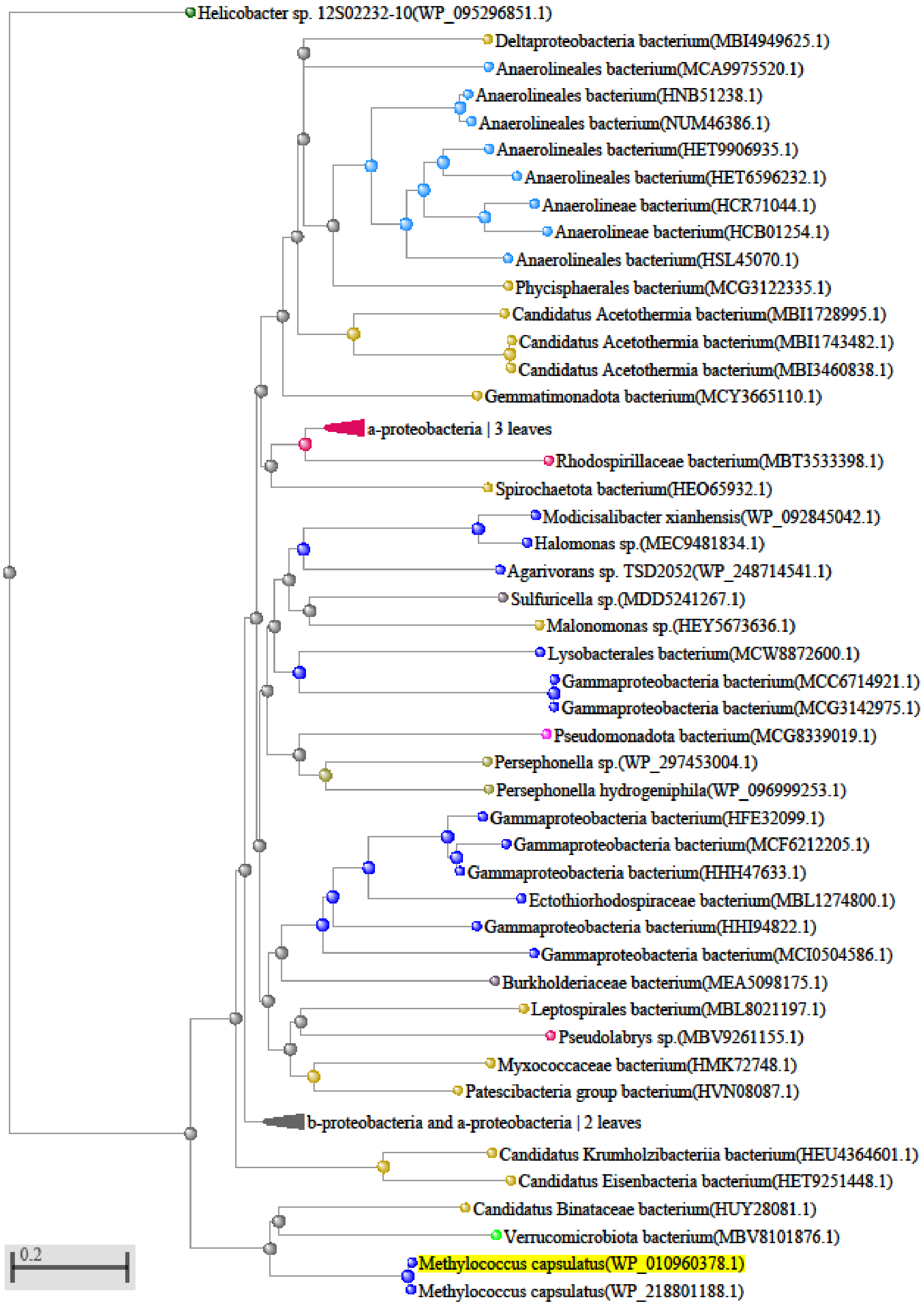

Figure S7. Cladogram of the *M. capsulatus* Bath CA2 (MCA1080) top 50 orthologs identified by Blastp. The *M. capsulatus* Bath query sequence is highlighted, Protein accession numbers are indicated in parentheses. *Alphaproteobacteria* (red nodes); Betaproteobacteria (purple nodes); *Gammaproteobacteria* (dark blue nodes); *Epsilonproteobacteria* (dark green nodes); green non-sulfur bacteria (light blue nodes); bacteria (yellow nodes); Proteobacteria (pink nodes); Verrucomicrobia (green nodes); Aquificales (gold nodes). Scale represents substitutions per site.

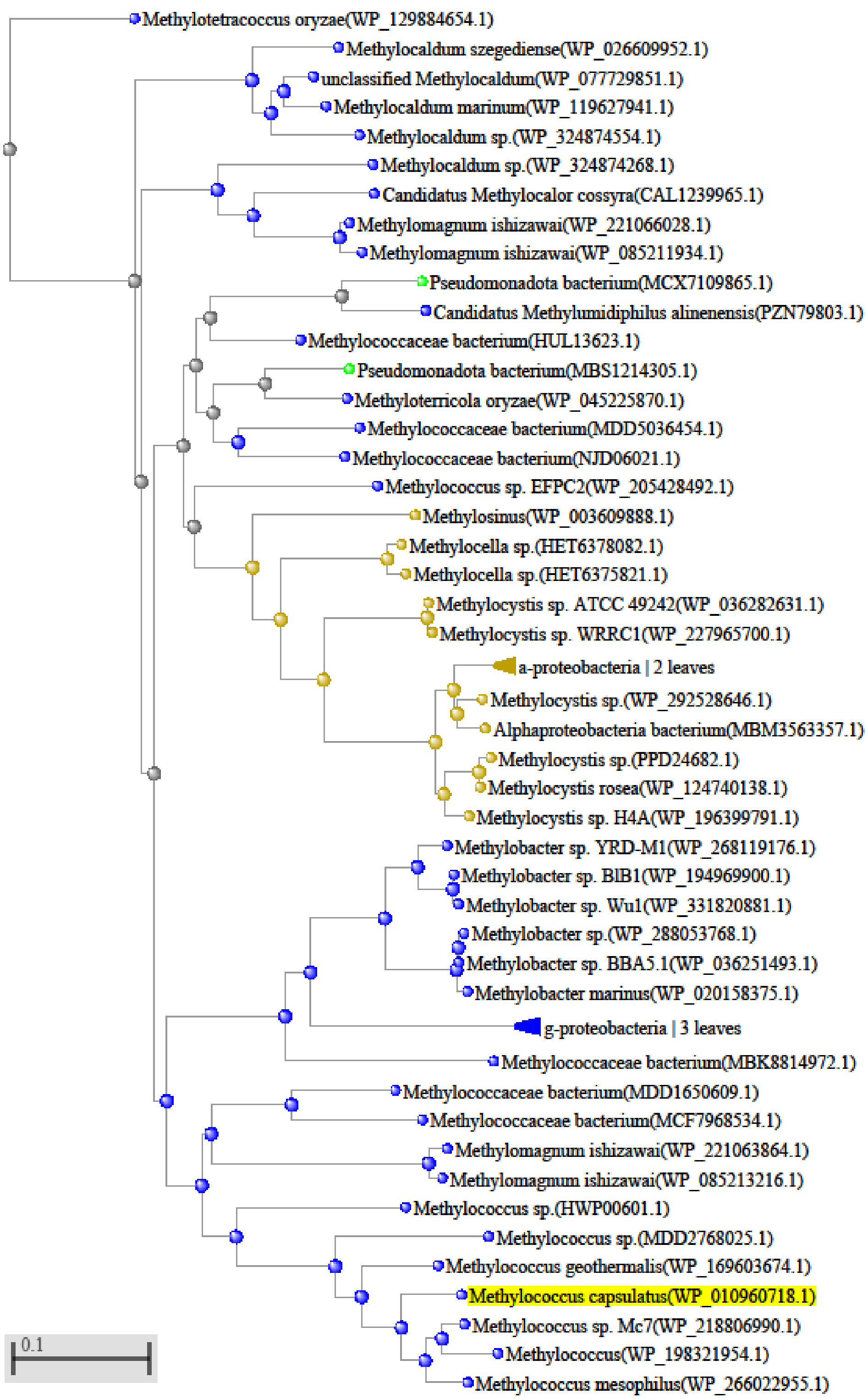

Figure S8. Cladogram of the *M. capsulatus* Bath CA3 (MCA1442) top 50 orthologs identified by Blastp. The *M. capsulatus* Bath query sequence is highlighted, Protein accession numbers are indicated in parentheses. *Alphaproteobacteria* (yellow nodes); *Gammaproteobacteria* (dark blue nodes); Proteobacteria (yellow nodes). Scale represents substitutions per site.

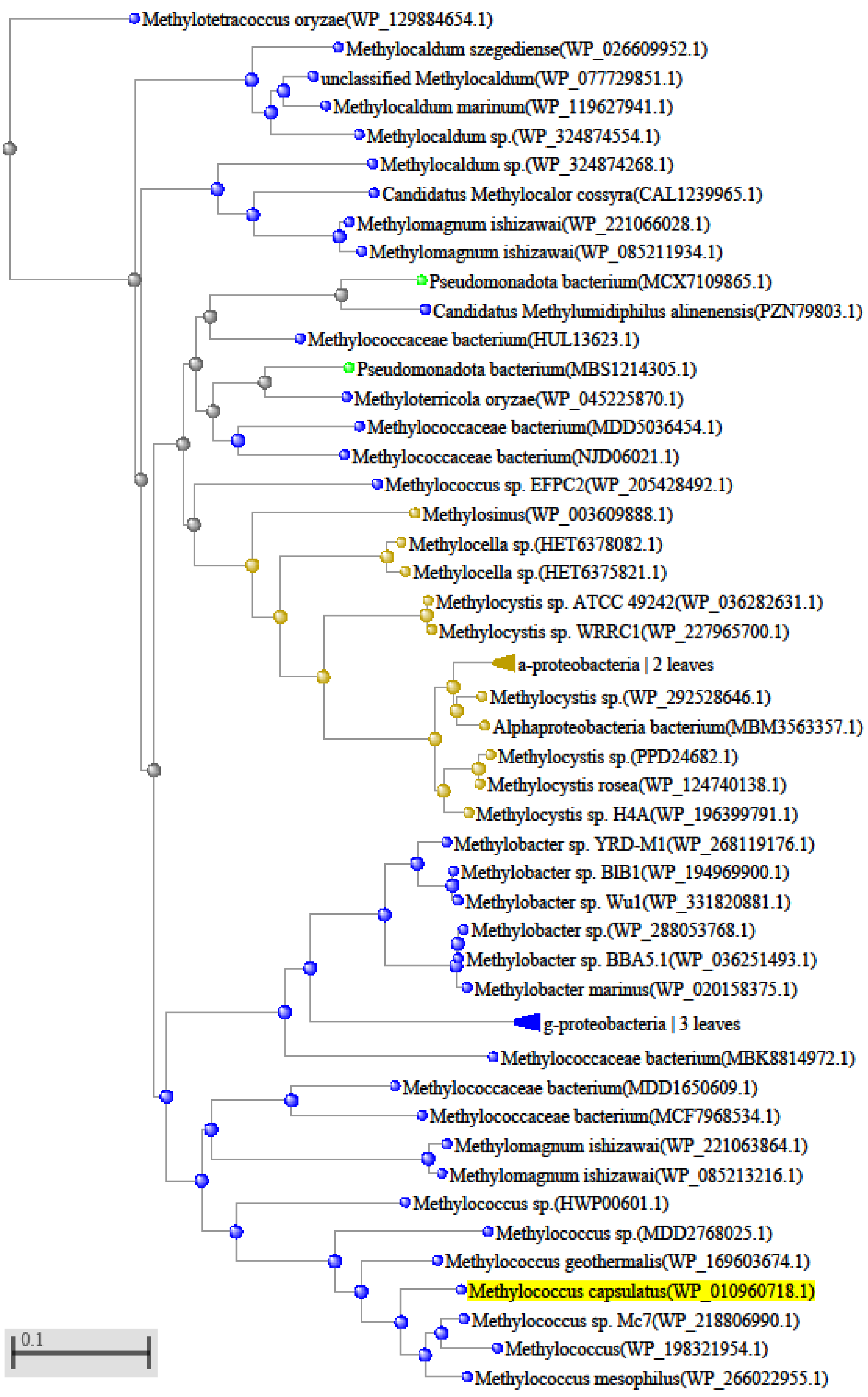

Figure S9. Cladogram of the *M. capsulatus* Bath CA4 (MCA1665) top 50 orthologs identified by Blastp. The *M. capsulatus* Bath query sequence is highlighted, Protein accession numbers are indicated in parentheses. *Alphaproteobacteria* (yellow nodes); *Gammaproteobacteria* (dark blue nodes); Proteobacteria (green nodes). Scale represents substitutions per site.

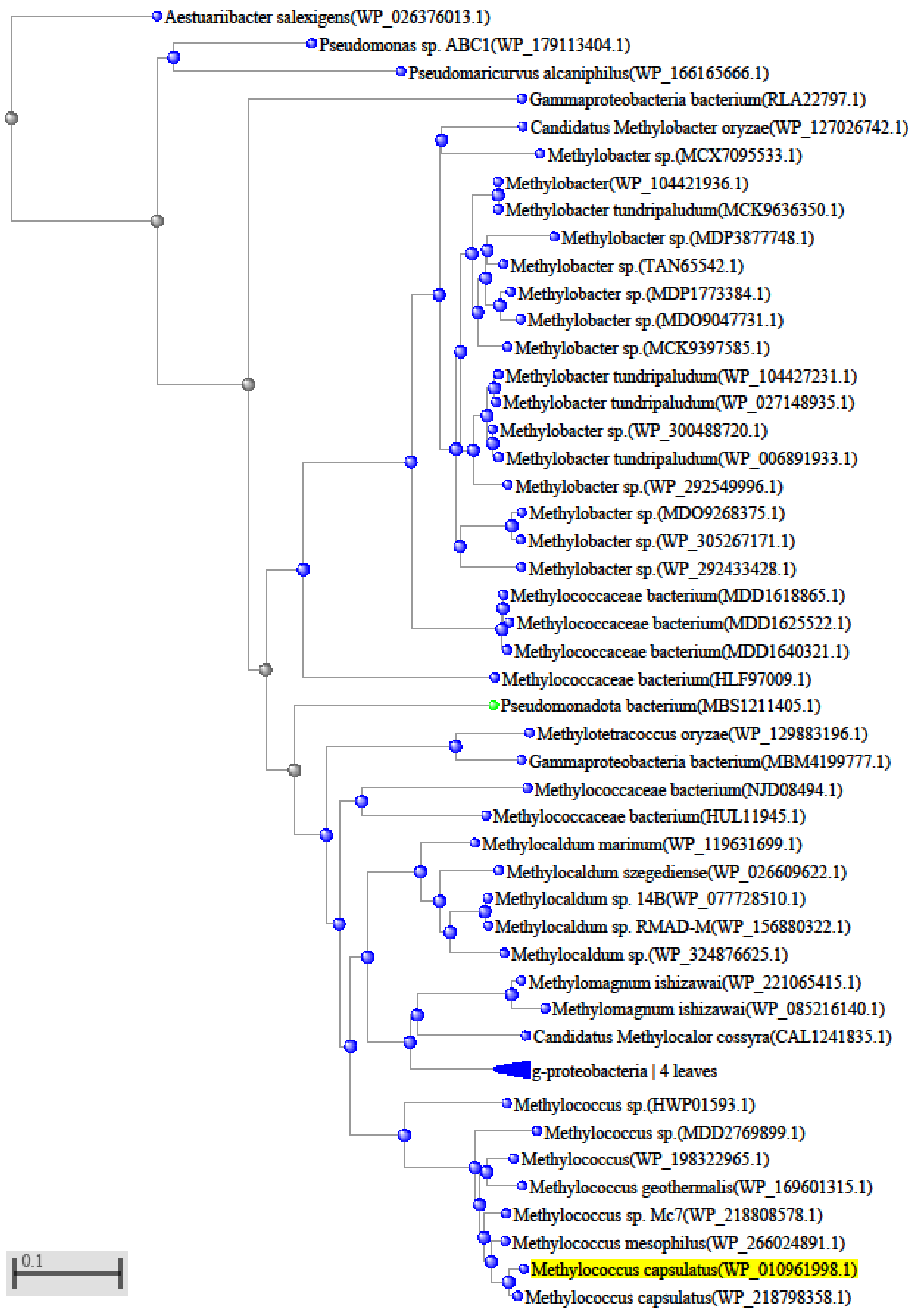

Figure S10. Cladogram of the *M. capsulatus* Bath CA5 (MCA2797) top 50 orthologs identified by Blastp. The *M. capsulatus* Bath query sequence is highlighted, Protein accession numbers are indicated in parentheses. *Gammaproteobacteria* (dark blue nodes); Proteobacteria (green nodes). Scale represents substitutions per site.

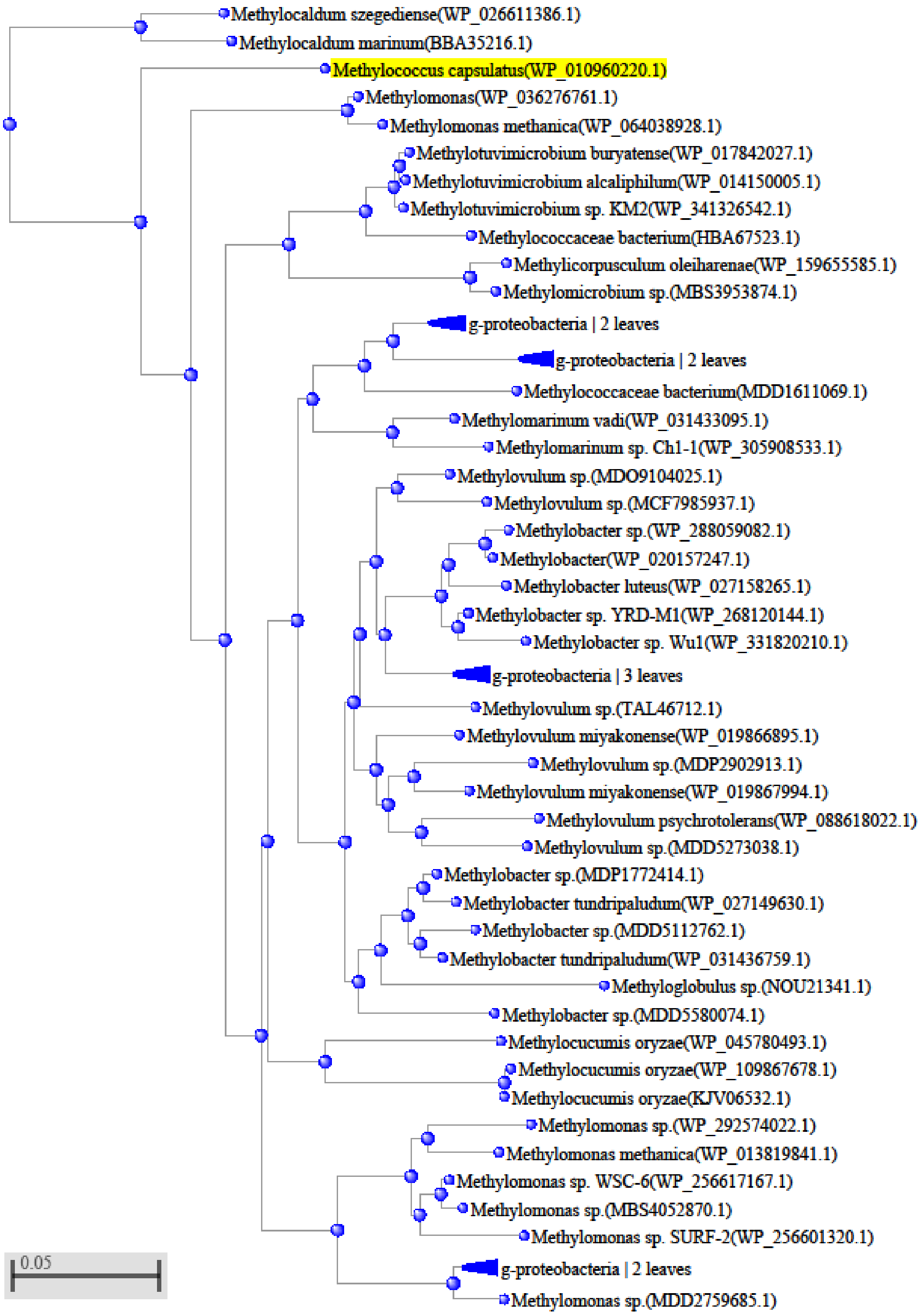

Figure S11. Cladogram of the *M. capsulatus* Bath CA1 (MCA0910) top methanotroph orthologs identified by Blastp. The *M. capsulatus* Bath query sequence is highlighted, Protein accession numbers are indicated in parentheses. *Gammaproteobacteria* (dark blue nodes). Scale represents substitutions per site.

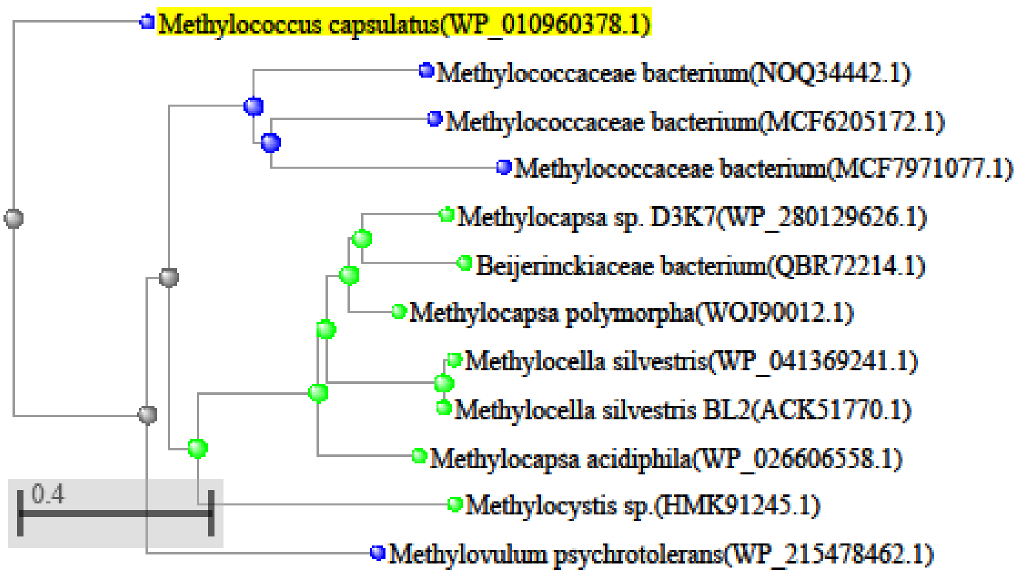

Figure S12. Cladogram of the *M. capsulatus* Bath CA2 (MCA1080) methanotroph orthologs identified by Blastp. The *M. capsulatus* Bath query sequence is highlighted, Protein accession numbers are indicated in parentheses. *Alphaproteobacteria* (green nodes); *Gammaproteobacteria* (dark blue nodes). Scale represents substitutions per site.

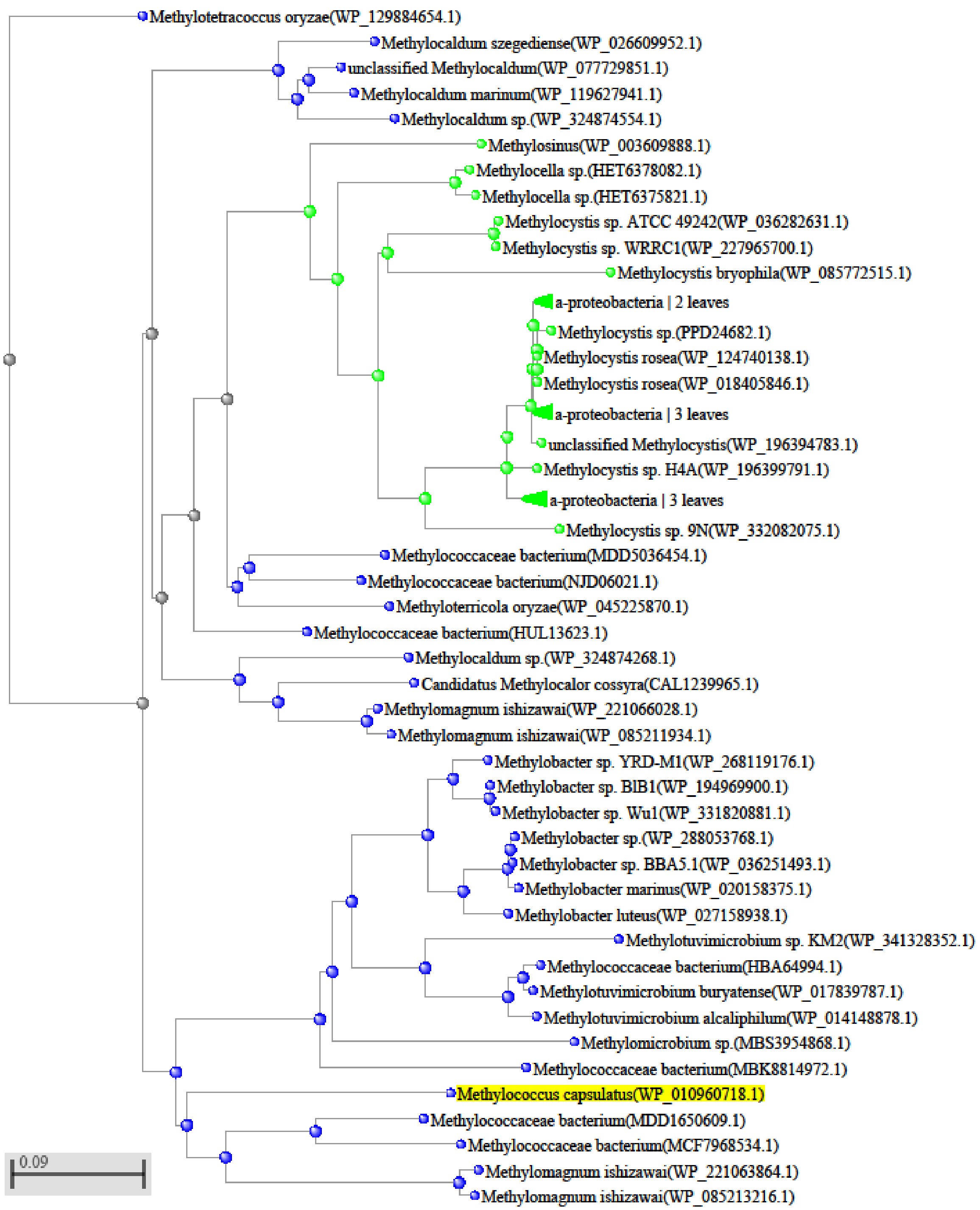

Figure S13. Cladogram of the *M. capsulatus* Bath CA3 (MCA1442) top methanotroph orthologs identified by Blastp. The *M. capsulatus* Bath query sequence is highlighted, Protein accession numbers are indicated in parentheses. *Alphaproteobacteria* (green nodes); *Gammaproteobacteria* (dark blue nodes). Scale represents substitutions per site.

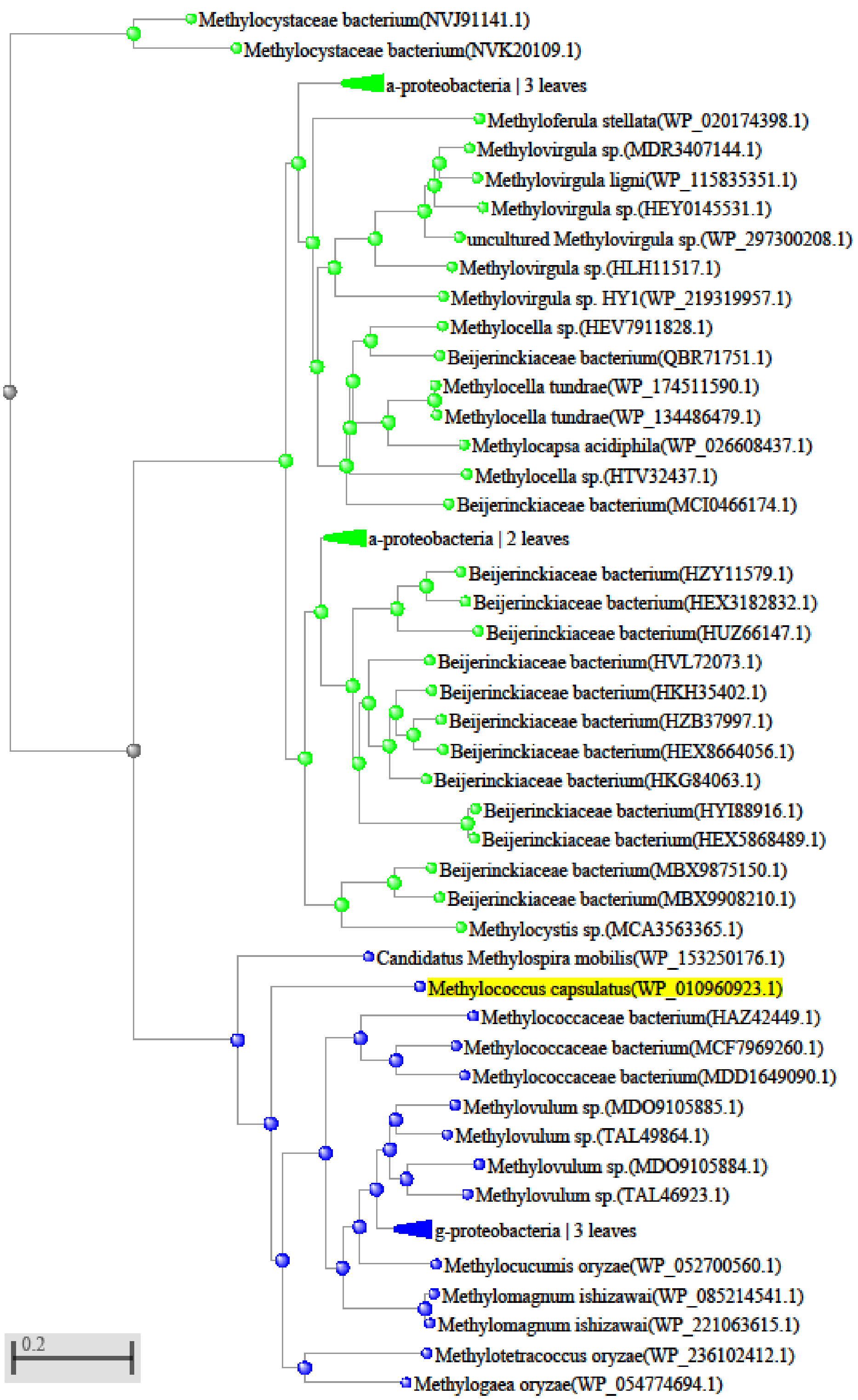

Figure S14. Cladogram of the *M. capsulatus* Bath CA4 (MCA1665) top methanotroph orthologs identified by Blastp. The *M. capsulatus* Bath query sequence is highlighted, Protein accession numbers are indicated in parentheses. *Alphaproteobacteria* (green nodes); *Gammaproteobacteria* (dark blue nodes). Scale represents substitutions per site.

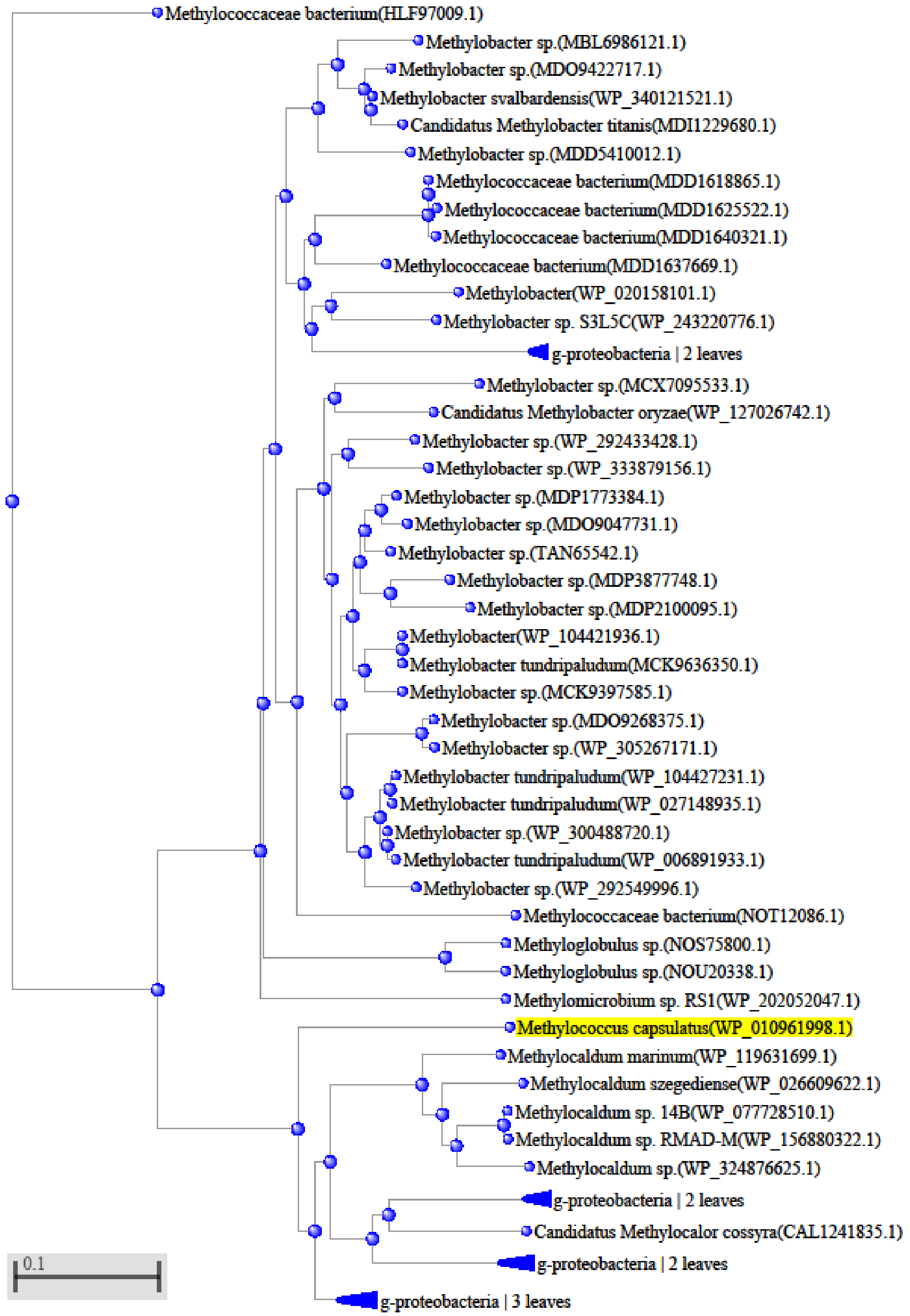

Figure S15. Cladogram of the *M. capsulatus* Bath CA5 (MCA2797) top methanotroph orthologs identified by Blastp. The *M. capsulatus* Bath query sequence is highlighted, Protein accession numbers are indicated in parentheses. *Gammaproteobacteria* (dark blue nodes). Scale represents substitutions per site.

A

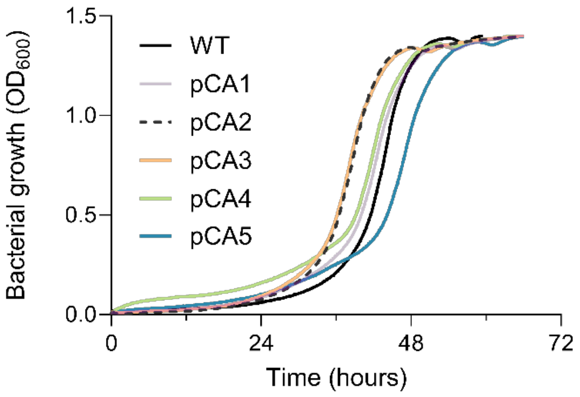

B

Strain	Specific Growth Rate (h^{-1})
**WT**	0.094 ± 0.001
**pCA1**	0.101 ± 0.001
**pCA2**	0.112 ± 0.010
**pCA3**	0.102 ± 0.002
**pCA4**	0.087 ± 0.006
**pCA5**	0.102 ± 0.001
**pCA2-3**	0.109 ± 0.016

Figure S16. A) High resolution carbonic anhydrase (CA) overexpression strain cultivation and (B) maximum specific growth rates during logarithmic growth. The growth curve data is the mean of two biological replicates. Data in B represent the mean ± SD of two biological replicates.

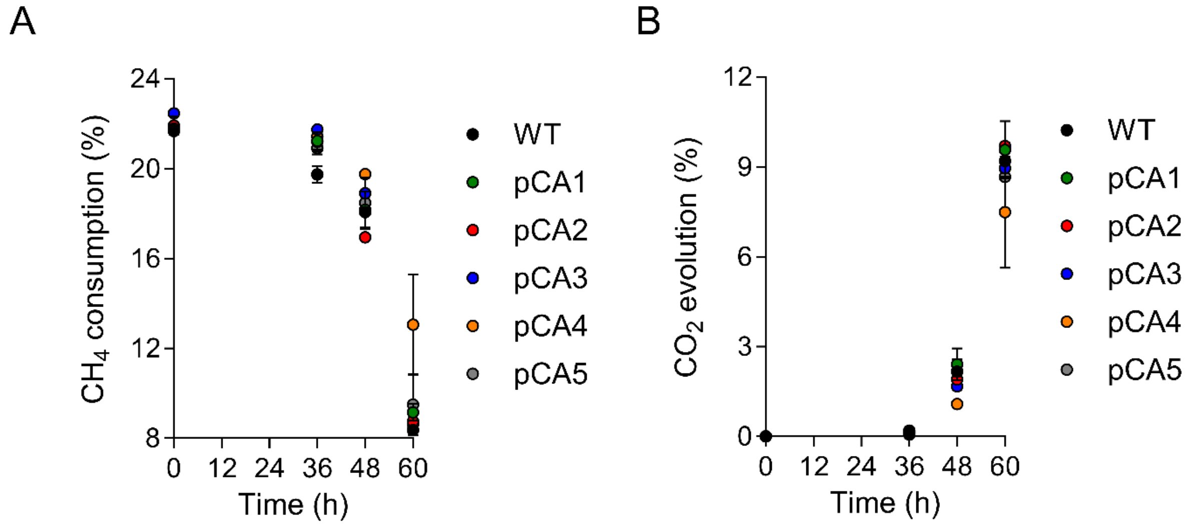

Figure S17. A) CH_4 consumption and (B) CO_2 evolution during cultivation of the carbonic anhydrase (CA) overexpression strains.
